# Longitudinal analysis of academic stress and its effects on salivary cortisol, alpha-amylase, and academic outcomes: Study protocol

**DOI:** 10.1371/journal.pone.0315650

**Published:** 2024-12-20

**Authors:** Juan Luis Castillo-Navarrete, Alejandra Guzmán-Castillo, Claudio Bustos

**Affiliations:** 1 Facultad de Medicina, Departamento de Tecnología Médica, Universidad de Concepción, Concepción, Chile; 2 Programa de Neurociencia, Psiquiatría y Salud Mental, NEPSAM, Universidad de Concepción, Concepción, Chile; 3 Facultad de Medicina, Programa Doctorado en Salud Mental, Universidad de Concepción, Concepción, Chile; 4 Facultad de Medicina, Departamento de Ciencias Básicas y Morfología, Universidad Católica de la Santísima Concepción, Concepción, Chile; 5 Facultad de Ciencias Sociales, Departamento de Psicología, Universidad de Concepción, Concepción, Chile; PLoS ONE, UNITED STATES OF AMERICA

## Abstract

**Introduction:**

Academic stress is a prevalent problem among university students, affecting both their psychological well-being and academic performance. This study aims to investigate the mediating roles of biological and psycho-behavioural variables in the relationship between academic stress and academic performance over the course of a semester. Through a longitudinal approach and using accessible data collection technologies, the results will enable the design of effective interventions to mitigate the impact of academic stress.

**Hypotheses:**

(i) Biological variables related to academic performance will mediate the relationship between academic stress and students’ academic performance. (ii) Psycho-behavioural variables will also act as mediators in this relationship, impacting academic performance differently.

**General objective:**

To explore the mediating roles of biological and psycho-behavioural variables in the relationship between academic stress and academic performance over the course of a university semester.

**Design:**

A longitudinal non-experimental observational design will be applied. Data will be collected in three assessment cycles, each consisting of three consecutive weeks during the academic semester.

**Participants:**

A sample of 160 undergraduate students from the Faculty of Medicine of the University of Concepción will be included. Students will be recruited on a voluntary basis through social networks and student associations. Students under psychological or pharmacological treatment will also be included to more representatively reflect the student reality and to ensure the ecological validity of the study.

**Biological and psycho-behavioural data collection:**

Participants will answer electronic questionnaires on academic stress and psycho-behavioural variables three times a week via the REDCap platform. In addition, smart devices will be used to continuously collect biological data such as heart rate, oxygen saturation, and sleep patterns. Students will also collect saliva samples three times a week to measure cortisol levels, and alpha-amylase enzyme activity.

**Statistical analysis:**

(i) Descriptive analysis of variables will be performed using measures of central tendency and dispersion for continuous variables and frequencies and percentages for categorical variables. (ii) Bivariate and multivariate analyses will be conducted to compare groups. (iii) Random intercept cross-lagged models will be used to assess the direction and reciprocal effects between variables over time. To analyze mediations, structural models (SEM) will be applied, considering biological and psycho-behavioural variables as mediators.

**Expected results:**

It is anticipated that (i) biological variables, such as cortisol and salivary alpha-amylase, will play a significant mediating role in the relationship between academic stress and academic performance, particularly towards the end of the semester. (ii) psycho-behavioural variables will also have a mediating effect, with different impacts on academic performance depending on the level of stress experienced. The use of accessible technologies and non-invasive methods such as saliva sample collection will provide a replicable model for future research.

## Introduction

Mental health is essential to everyone’s well-being, and this is especially true for university students. Today, many young people embark on higher education with the hope of gaining a degree and improving their job prospects. However, this path can be fraught with obstacles that, if not managed properly, may lead to mental health problems that affect both academic performance and retention in university. Academic stress is one of the main factors that trigger these problems. When students are under constant pressure to meet the demands of their studies, such as homework, deadlines, exams, and peer competition, it is easy for anxiety, depression, sleep disorders, and even problems in interpersonal relationships to appear, undermining their well-being and academic performance. Thus, it is essential to pay attention to the mental health of university students. If these problems are not addressed promptly, the risk of young people dropping out of their studies increases considerably. Emotional exhaustion and a feeling of not being able to cope with the demands can lead them to abandon their dreams, which has a negative impact on their future both personally and professionally.

Addressing the phenomenon of stress is a complex challenge, especially in the university student population that faces a particular type of stress known as academic stress. Although several studies have been conducted on this topic, much remains unknown about its effects. This proposal focuses on exploring how academic stress impacts different physiological and psycho-behavioral aspects of university students’ academic performance. To contextualize this proposal, the following provides information on three key areas: (i) academic stress, (ii) perceived stress, and (iii) physiological and psycho-behavioral domains related to academic performance.

### Academic stress

Stress is a complex response involving neuroendocrine, immunological, and behavioral aspects, and is activated when an organism faces any kind of demand [1]. Depending on the nature of the stressor, this adaptive response can be acute or chronic [1–3]. In acute stress situations, increases in heart rate, blood pressure, respiratory rate, blood glucose levels, and coagulation factors are observed [4,5]. After this initial phase, the organism enters a coping phase, followed by a relaxation phase that allows a return to physiological homeostasis [3–5]. When stress becomes chronic, the organism also goes through a phase of alertness and a stage of resistance. However, if exposure to the stressor persists, it reaches a phase of exhaustion, characterized by the organism’s inability to maintain homeostatic balance. This imbalance can trigger various pathophysiological phenomena, such as an increase in proinflammatory cytokines, and acute-phase proteins, structural changes in the hippocampus, and multiple hormonal alterations [6–9].

The stress response activates several neurophysiological systems, that operate through different mechanisms [10]. The two main systems involved are the hypothalamic-pituitary-adrenocortical (HPA) axis, and the autonomic nervous system (ANS). The HPA axis is a complex neuroendocrine system that plays a crucial role in the regulation of mood, and cognitive functions. Through stimulatory pathways and feedback loops, the HPA regulates the production of stress-related hormones such as cortisol [5,11].

Stress also affects the sleep cycle, and partial or total loss of sleep can further alter the HPA response, resulting in increased cortisol levels [11–13]. When a person faces physical or mental stress, large amounts of cortisol are released, triggering alert responses that increase blood glucose levels and alter mental reactions. This process makes cortisol a biomarker closely related to stress levels [11,14–16]. In situations of chronic stress, the constant activation of HPA leads to increased cortisol production, as well as neuroinflammatory cytokines, among other effects [11,17]. The ANS stress response is manifested through the release of catecholamines (epinephrine and norepinephrine) by chromaffin cells in the adrenal medulla, innervated by the cholinergic system [10]. These catecholamines generate a number of physiological responses, such as increased heart rate, blood pressure, and salivary amylase production, preparing the body for the ‘fight or flight’ response to the stressor [18–20].

When a stressful experience arises in the context of an educational process, it is referred to as academic stress. This type of stress can manifest itself even in primary school students and tends to intensify as students’ progress through their education, reaching its peak during the university stage [21,22]. Higher education is particularly prone to high levels of academic stress due to heavy workloads and periods of high demand that require a great deal of adaptive effort on the part of students. As a result, they may experience burnout, disinterest in studying and even difficulties in coping with the challenges of their academic environment [23].

Despite its frequent use, the term ‘academic stress’ is often handled imprecisely, and its true scope and limitations may not be fully understood. Confusion is increased by the variety of terms used to describe similar experiences, such as student stress, university stress, burnout, school stress and exam stress. The lack of clear conceptualization and the tendency to focus on specific stressors and symptoms make these terms two-dimensional and limited in capturing the complexity of stress. Moreover, these concepts are often intertwined with other multidimensional constructs such as anxiety and psychological vulnerability, and the diversity of assessment instruments used further complicates their understanding [24,25].

Consequently, a multidimensional approach is required to understand and address academic stress. However, the measurement of stress in the university context has been based on instruments that provide simple and poorly contextualized measures. There are also instruments that assess stressful situations related to academic, family and economic aspects, such as the ‘Student-Life Stress Inventory’ [26], ‘Undergraduate Sources of Stress Questionnaire’ [27], ‘Academic Expectation Stress Inventory’ [28] and ‘College Student Stress Scale’ [29]. Others focus on the stressful potential of different academic conditions, such as the Academic Stressors Scale of the Academic Stressor Questionnaire (ECEA) [30–32].

Arturo Barraza Macías, in 2007, proposed a theoretical model with a more comprehensive and processual perspective of academic stress, analysing stressful situations, psychophysiological responses and coping strategies, and defining academic stress as a systemic adaptive and psychological process, with three moments: coping with demands considered stressors, systemic imbalance manifested in symptoms and coping actions to restore the balance [24], being able to identify three systemic-processual components: stressor stimuli, symptoms indicative of the imbalance and coping strategies. Based on this theoretical model, Barraza developed, in Mexico, a self-descriptive psychometric instrument, the SISCO inventory of academic stress, based on a 3-factor structure for the dimensions of stressors, symptomatology, coping and complete instrument, respectively [25].

The SISCO-II—an updated version of this inventory, maintains the dimensions of stressors, symptomatology, and coping, but now breaks down symptomatology into physical, psychological and social-behavioral reactions [23]. In addition, recent advances have established norms for the SISCO-II, allowing for a more standardized and accurate assessment of stress levels in university students, both individually and collectively [33]. Unlike other instruments that have been adapted from general stress models, the SISCO-II has been developed from the beginning with a focus on academic stress, which ensures its relevance and validity in this specific context. This particularity makes it an essential tool for stress research in higher education, providing a more contextualized and specific view of the stress dynamics faced by students.

Since academic stress is not limited simply to the presence of external demands, but also includes how the student perceives and copes with them, it is crucial that the assessment of academic stress considers not only stressors and symptomatology, but also the student’s subjective perception of these situations. In this context, the term ’perceived stress’ in university students refers to the subjective perception of an excessive burden of academic, social or personal demands that exceed the student’s ability to cope. This perception generates a negative emotional response, that affects their psychological, emotional and physical well-being [34,35].

It is important to note that perceived stress is subjective and varies between individuals, meaning that what may be stressful for one student may not be stressful for another. This type of stress in university students can have various consequences, such as decreased motivation, negatively affecting academic performance and emotional well-being. In addition, it can increases anxiety levels and create family problems, especially among those with limited financial resources. Students who experience high levels of perceived stress may also manifest physical symptoms such as fatigue, tiredness, and lack of energy, putting them at greater risk of developing somatic disorders.

It is important to note that, from an academic perspective, grades and the pressure generated around them can be a significant factor in the genesis of academic stress. Anxiety about obtaining good results and fear of failure can increase the perception of stress and have a negative impact on students’ mental and emotional well-being, even more so when grades are considered a key indicator of academic success, and students often face enormous pressure to obtain outstanding results in order to secure future opportunities, such as employment or graduate studies [36]. Grades, in addition to being indicators of academic performance, can help identify those at risk of dropping out [36]. The decision to drop out of higher education is a complex phenomenon, preceded by the intention to do so and is related to both individual and environmental factors [36,37]. Dropping out has been associated with dysfunctional self-regulation of learning and lack of cognitive skills in students [36,38].

As a result, over the course of an academic semester, university students face an increased load of academic challenges, which impacts various aspects related to academic performance, encompassing both physiological elements and those directly linked to the academic environment. Among the physiological components affected, those related to the HPA axis and the ANS stand out. The alterations linked to the HPA axis include changes in cortisol levels and sleep patterns, while those associated with the ANS include changes in heart rate, blood pressure and salivary amylase levels. For the purposes of this project, these physiological parameters will be defined as biological variables associated with academic performance (BADA).

In turn, in the academic domain, a series of psycho-behavioural behaviours can be identified that result in alterations in academic self-efficacy to manage stress, self-regulation of learning, academic satisfaction, rumination, procrastination and engagement (among others). For this project, these behaviours will be defined as psycho-behavioural variables associated with academic performance (PADA).

### HPA axis and stress

As previously mentioned, the HPA axis is a complex neuroendocrine system involving interactions between the hypothalamus, pituitary, and adrenal glands. This system is crucial for regulating various physiological functions in the body. When faced with a stressor, the hypothalamus releases corticotropin-releasing hormone (CRH), which stimulates the pituitary to produce and release adrenocorticotropic hormone (ACTH). ACTH, at the adrenal level, triggers the release of cortisol, a key glucocorticoid in the regulation of energy metabolism, immune response, water-electrolyte balance, cardiovascular homeostasis, and modulation of the inflammatory and stress response, thus contributing to the overall physiological adaptation of the organism [5]. However, prolonged or chronic activation of the HPA axis due to stress can have negative health effects, such as sleep disturbances, mood disturbances, cardiovascular problems, and immunosuppression [11].

Cortisol is a hormone produced by the adrenal cortex as a result of activation of the HPA axis. It follows a circadian pattern, reaching its peak release level 30–60 minutes after waking, and gradually decreases throughout the day, with its lowest levels at night [39,40]. In the presence of a stressor, the HPA axis is activated, releasing cortisol into the blood, whose peak in saliva is reached approximately 25–30 minutes later [40]. Cortisol levels are associated with the degree of stress and anxiety in stressful situations [41–43]. Reference ranges for cortisol levels in blood and saliva in healthy individuals are 30–160 ng/mL and 1–1.6 ng/mL, respectively [44–46].

The use of salivary cortisol in stress studies has grown due to the ease of collection and its non-invasive nature, allowing multiple samples to be taken throughout the day [39,44,47–49]. The linear relationship between blood and saliva cortisol concentrations is well documented [39] and salivary levels are independent of salivary flow velocity. Furthermore, the time lag between changes in plasma and saliva is only 1 to 2 minutes, ensuring a precise correspondence between both markers [48–50].

One of the most studied cortisol responses is the cortisol awakening response (CAR), which reflects the significant increase in cortisol in the first 30–45 minutes after awakening. This marker is used to assess the dynamic function of the HPA axis and is related to the body’s ability to adapt to stress [44,47,51]. To analyse cortisol levels over time, several strategies based on the calculation of the area under the curve (AUC) are used. Among the most common are the area under the curve with respect to increase (AUCi), which measures the increase in cortisol following a stimulus, and the area under the curve with respect to baseline (AUCg), which measures the total level of cortisol secreted over a specific time period [49,52]. These strategies provide a broad view of the dynamics of cortisol secretion in response to different stimuli.

The HPA axis, circadian rhythm and sleep cycles are closely related, as cortisol secretion follows a diurnal pattern that influences the consolidation of the sleep-wake cycle and the body’s adaptation to stressful situations [11]. Wakefulness and sleep are physiological states that alternate in a cyclical manner in humans [53]. Sleep deprivation or lack of adequate sleep can cause various disturbances, such as problems with attention, perception and memory, as well as irritability and stress, which affect higher cognitive functions and impair the ability to perform daily activities effectively [53,54].

Stress experienced during the day is one of the factors that can alter the quantity and quality of sleep [55]. This relationship is bidirectional, as sleep problems can increase stress, and stress, in turn, can cause disturbances in the sleep cycle [56,57]. Sleep is divided into two main stages: slow-wave sleep (with its respective phases) and rapid eye movement (REM) sleep, which alternate in an ultradian rhythm throughout the night [54,56,57]. Adults experience 4–6 ultradian cycles per night, with each cycle lasting approximately 90 minutes on average. Decreased sleep hours, delayed bedtimes, or early awakenings negatively affect students’ learning ability, neurobehavioral functioning, and thus academic performance [53–57].

### ANS and stress

The response to stress through ANS affects both blood pressure and heart rate, generating an increase in both parameters, especially in students experiencing academic stress [58]. This increase is related to anxiety levels in specific academic situations, such as the moments before an assessment, showing variations throughout the day [58–60]. While temporary increases in blood pressure and heart rate in response to academic stress are not necessarily detrimental in the short term, chronic stress and prolonged exposure to high levels of stress may have long-term negative effects on students’ cardiovascular health [58,60].

Salivary alpha-amylase (sAS) has emerged as a biomarker relevant to the activity of the autonomic nervous system (ANS), specifically the sympathetic-adrenal-medullary system (SAM), and has been shown to be a useful tool for assessing the impact of stress on the body [42,61]. sAS, synthesised mainly by the parotid gland, is one of the key components of saliva, playing roles in enzymatic digestion and antibacterial protection, as well as acting as a marker sensitive to sympathetic activation [42]. The release of sAS is regulated by the sympathetic and parasympathetic nervous systems, responding to the action of neurotransmitters such as noradrenaline, which activates α-adrenergic and β-adrenergic receptors, triggering a series of events that culminate in its excretion [61,62].

Studies have shown that, during episodes of acute stress, anxiety, and chronic stress, sAS excretion increases due to overactivation of the autonomic system, making it a reliable biomarker for measuring sympathetic activity [42,63]. In addition, sAS exhibits a diurnal profile characterised by lower levels during the first 30 minutes after awakening, followed by a steady increase that peaks in the late afternoon [42,61]. This diurnal pattern may be altered under conditions of chronic ANS dysregulation, with an attenuated response upon awakening and hypersecretion during the day [61].

Measurement of sAS is especially useful in research settings, as it provides a non-invasive, reliable, and pain-free assessment of ANS functioning, thus facilitating sampling in sensitive populations, such as university students under academic stress [42]. Unlike serum α-amylase, which is derived from pancreatic secretion and whose levels are unrelated to those of sAS, the determination of this enzyme in saliva can be performed by routine methods, such as spectrophotometric or dry chemistry techniques, making it an accessible marker for the study of stress [10,59,62].

In addition to its role as a biomarker of autonomic nervous system (ANS) activity, salivary alpha-amylase (sAS) has also been studied in relation to its interaction with the hypothalamic-pituitary-adrenal (HPA) axis. Research has shown that, in situations of psychological stress, sAS shows a more rapid and sensitive response compared to cortisol, making it an efficient marker for assessing the impact of stress in real time [64,65]. The stress response includes both a catecholamine component, associated with the sympathetic-adrenal-medullary system (SAM), and a slower response linked to the HPA axis. From this perspective, the sAS is initially activated through the SAM, whereas cortisol is released at a later stage [61]. This rapid response of the sAS makes it particularly useful for assessing acute stress fluctuations in academic settings, where students may experience abrupt changes in their stress levels. Additionally, it has been observed that under conditions of chronic stress, such as in patients with post-traumatic stress disorder (PTSD), the sAS exhibits an altered diurnal rhythm, which could be relevant when assessing the impact of academic stress over the course of a semester [66,67]. Table 1 summarises the physiological components, the parameters involved and their relationship to stress and impact on academic performance.

**Table 1:** Physiological components, involved parameters, and their relationship with stress and its impact on academic performance.

Physiological Components	Parameters	Relationship with Stress	Impact on Health and Academic Performance
HPA Axis	Cortisol levels and sleep patterns	Regulation of the stress response	Negative effects due to chronic activation
ANS	Heart rate, blood pressure, and salivary amylase levels	Physiological response to stress	Long-term risk to cardiovascular health
Circadian rhythm and sleep	Sleep-wake cycles	Influence on adaptation to stress	Impact on cognitive functions and learning ability

As for the psycho-behavioural patterns associated with academic performance (PADA), it is essential to explore them further in order to understand more precisely their impact on performance in the context of academic stress.

Academic self-efficacy to manage stress refers to students’ perception of their ability to organise and execute actions to achieve optimal academic performance, especially in high-demand situations [68]. This perception of self-efficacy, influenced by previous achievements, observation of others, external persuasion and emotional states, is determinant factor for the effective management of academic tasks and the reduction of associated stress [69]. High self-efficacy not only fofosters motivation but alsoositively affects student perception and effort, which contributes significantly to academic success. It is crucial to understand that self-efficacy is not based on the resources possessed by the individual, but on the perceived ability to use them effectively, which is especially important in academic contexts involving high pressure and emotional demand [68,69].

Self-regulation of learning (SRL) is an active process that enables students to establish clarity in their academic goals and effectively regulate their cognitions, motivations, and behaviours throughout the learning process [70]. This holistic approach to academic development includes task planning, persistence in the face of difficulties, and adjusting strategies to achieve success, and is essential for high academic performance [36]. ARA is characterized by a three-phase cycle: disposition, performance, and evaluation, in which the student plans, executes and reflects on their actions with the aim of continuously improving their performance [70,71]. This capacity for self-regulation is a key factor for success in demanding academic environments, as it enables students to cope more effectively with the demands and stress associated with the educational environment.

Academic satisfaction refers to the perceptions of well-being that students experience when performing activities linked to their academic role [72]. This concept is closely related to academic progress, beliefs in one’s own abilities, positive expectations and perceived self-efficacy. In addition, academic satisfaction also reflects students’ degree of satisfaction in dimensions related to curricular, institutional, and personal aspects, as well as in their interactions with peers and teachers [72,73]. It is considered a cognitive-affective component that not only involves students’ enjoyment of their academic experience, but also allows them to assess the educational environment and adjust it to optimise student learning and well-being [74–76].

Procrastination refers to the recurrent tendency to postpone the start or completion of planned tasks within a given deadline, which often generates subjective discomfort [77,78]. In academia, procrastination is common among university students due to the deadlines of many academic activities. However, this behaviour not only reflects inefficient time management, but may also be related to self-regulation difficulties at cognitive, affective and behavioural levels [79]. Procrastination does not simply imply a lack of responsibility, but may be indicative of deeper problems in students’ ability to manage academic demands effectively, negatively affecting their academic performance [78].

Rumination refers to repetitive thoughts that, in its most studied form, depressive rumination, involve a tendency to persistently focus on negative moods. This cognitive process favours the predisposition, maintenance and intensification of depressive symptoms [80,81]. There are two main components of rumination: reflective rumination, which involves introspection about the depressive mood with the aim of understanding the causes of distress, and negative rumination, which refers to repetitive self-centred thoughts that passively re-experience depressive symptomatology [82,83]. Several studies have shown that negative rumination is more strongly associated with detrimental emotional consequences than reflective rumination, and is an important factor to assess in high-stress contexts, such as academia [82,84,85].

Academic engagement is closely related to students’ performance and well-being, reflecting an enthusiastic and optimistic attitude towards learning [86,87]. Students with high levels of engagement tend to show stronger intrinsic motivation, positive self-efficacy expectations, optimism and high self-esteem, which clearly differentiates them from those with low levels of academic engagement [87,88]. This engagement is key to academic and emotional success, with studies suggesting that women, in particular, tend to show greater energy and dedication towards their academic tasks compared to men [87].

### Problem and research question

University life poses a considerable challenge for many students, who must face a highly stressful and demanding environment that can negatively impact their academic performance in a number of ways. Often, these students are forced to work to support themselves financially, which increases their dependence on scholarships, family support or even indebtedness through national funding systems. As a result, they find themselves in an environment that is not only challenging, but also one of constant financial worries, which can affect both their physical and psychological well-being. Despite these difficulties, students must strive to maintain satisfactory academic performance, which is further complicated by the multiple stressors present in their environment.

In the current academic context, there has been a notable lack of research, especially longitudinal studies, that comprehensively and rigorously address the interaction of various stressors at university life and their influence on both the physiological well-being and psycho-behavioural patterns of students. Although the existing literature has explored the effects of certain academic stressors in isolation, there remains a marked paucity of studies that take a longitudinal and holistic approach to analyzing the combined impact of these factors on health and academic performance. The implementation of longitudinal studies would allow for a more accurate and contextualized assessment of the challenges faced by students in the contemporary academic environment, providing valuable information on trends and changes in students’ responses over time to academic stressors.

In this context, and in order to achieve a comprehensive assessment of the relationship between stress and student performance over time, a longitudinal analysis is indispensable. This approach will allow a deeper understanding of how academic stress affects university students during the course of an academic semester, and how it evolves over time. It will also make it possible to identify precisely the different physiological aspects and psycho-behavioural patterns that may be influenced by such stress, providing a more complete and dynamic view of its effects.

Therefore, the research question posed is: What is the mediating role of biological and psycho-behavioural variables associated with academic performance in the relationship between academic performance and academic stress over the course of a university semester? This question seeks to understand how biological and psycho-behavioural variables linked to academic performance influence the relationship between academic stress and students’ own performance. The concept of mediating role refers to the possibility that these variables explain, in whole or in part, the connection between the stress experienced in the academic context, and the results obtained in terms of academic performance.

## Method

**Fig 1:**
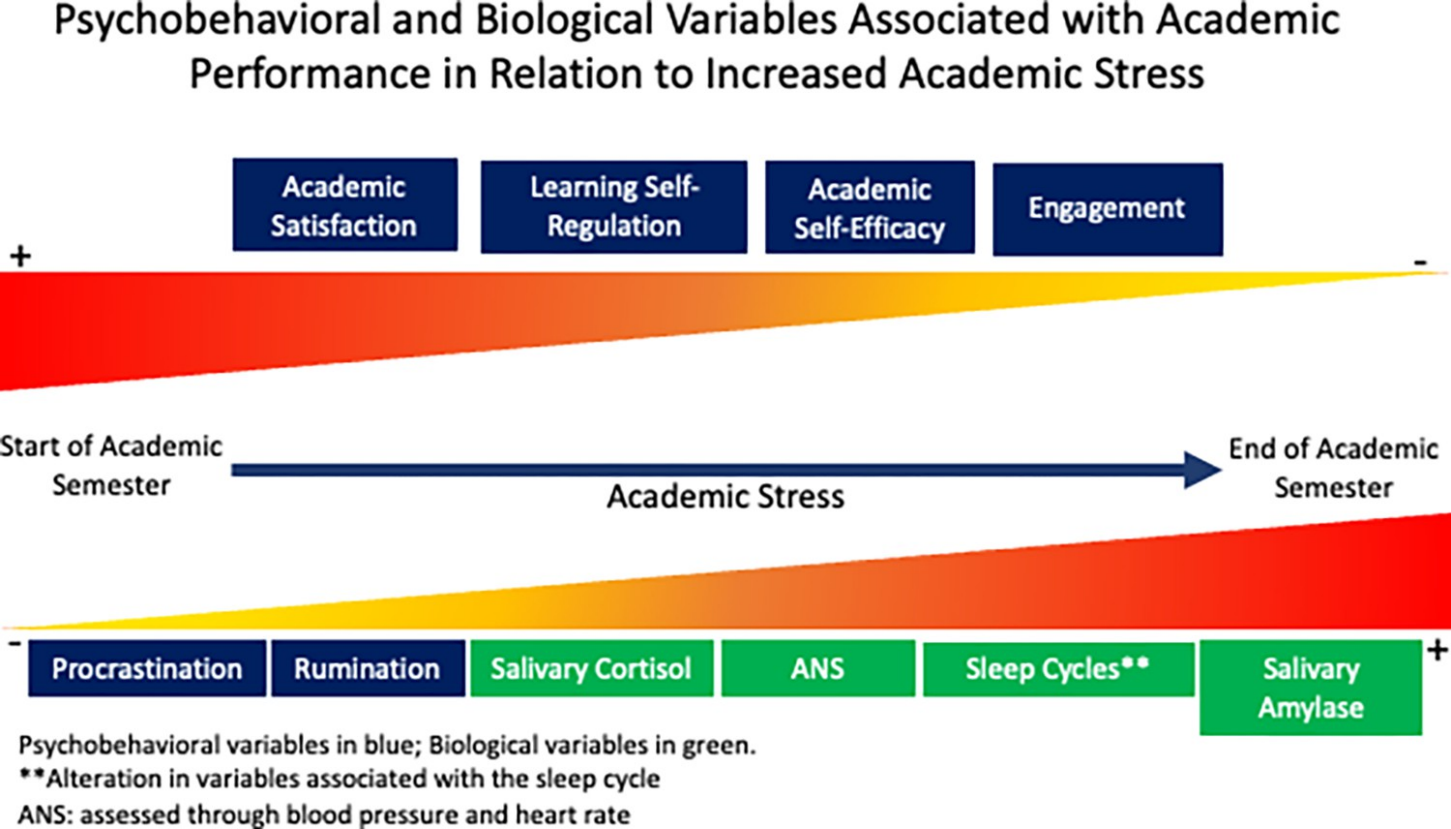
Biological and psychobehavioral variables associated with academic performance in relation to increased stress. It is expected that by the end of the academic semester, there will be an increase in procrastination, rumination, cortisol and salivary amylase levels, as well as in blood pressure, heart rate, and sleep pattern alterations. Likewise, a decrease is anticipated in academic satisfaction, learning self-regulation, academic self-efficacy, and engagement.

Considering that the research question focuses on analysing the mediating role of biological and psycho-behavioural variables associated with academic performance in the relationship between academic stress and students’ own performance throughout a university semester, it is pertinent to propose the following hypotheses (Fig 1): (i) Biological variables associated with academic performance will mediate the relationship between academic stress and students’ performance during the semester. (ii) Psycho-behavioural variables will also act as mediators in such a relationship during the same period.

### General objective

To analyse the mediating role of biological and psycho-behavioural variables associated with academic performance in the relationship between academic stress and university students’ performance over a semester.

### Specific objectives

(i) To longitudinally assess the level of academic stress and the biological and psycho-behavioural variables associated with academic performance of university students over the course of an academic semester. (ii) To determine the relationship between biological variables associated with academic performance and stress levels at university students over an academic semester. (iii) To determine the relationship between psycho-behavioural variables associated with academic performance and stress levels at university students over an academic semester. (iv) To determine the extent to which biological variables associated with academic performance mediate the relationship between stress and academic performance at university students over the course of an academic semester. (v) To determine the extent to which psycho-behavioural variables associated with academic performance mediate the relationship between stress and academic performance of university students over the course of an academic semester.

### Study design

A longitudinal non-experimental design is proposed. Data will be collected in three cycles during the semester, each lasting three consecutive weeks. Repeated measurements of stress-related biological and psycho-behavioural variables will be made on the same students throughout the semester, without deliberately manipulating the variables. The phenomena will be observed in their natural academic context and then analysed.

### Methodology

A quantitative methodology will be applied. Students will answer questionnaires on academic stress and PADA variables during each assessment cycle. A smart wearable device will collect continuous, real-time data on heart rate, oxygen saturation and sleep patterns (BADA variables). In addition, students will collect saliva samples three times per week to measure cortisol and salivary alpha-amylase levels, following a standardised protocol.

### Universe and sample

The universe of the study will be constituted by undergraduate students of the Faculty of Medicine of the Universidad de Concepción. A convenience sample will be obtained, composed of these same students. To calculate the sample size, a correlation matrix was used to simulate the psycho-behavioural variables (PADA), biological variables (BADA) and academic stress (Muthén, L. K., & Muthén, B. O. 2002). From this analysis, it was determined that the optimal sample would be 160 individuals, with a statistical power of 70% and a confidence level of 95%.

### Procedure

#### Participants

The study will involve undergraduate students from the Faculty of Medicine at the Universidad de Concepción (UdeC), with first-year students and those currently in professional practice excluded. All participants will voluntarily sign an informed consent form before joining the study.

#### Timing

Data collection will be organized into three measurement cycles during each academic semester, with each cycle lasting three consecutive weeks. Within these cycles, participants will complete questionnaires on academic stress and psycho-behavioral variables, while biological variables will be measured continuously using handheld devices to ensure data accuracy. Recruitment for participants will begin in the first week of the academic semester in early March 2025, which aligns with the start of classes in Chile. The study anticipates that data collection will be completed by the end of 2025 or, if needed, by the first semester of 2026. This timeline allows for data analysis to be completed by the end of 2026.

#### Recruitment

Students will be invited to participate via a mass email sent by the program leaders of each relevant degree program. Those interested in participating will provide their contact information to facilitate the distribution of handheld devices and saliva collection kits, along with the necessary instructions and training for proper self-collection of samples.

#### Inclusion criteria

Active students of the Faculty of Medicine, from second year onwards, under 25 years of age, who have not yet started their professional practice or internship, will be included in the study. Participants must freely express their willingness to participate by signing an informed consent form and be trained in self-collection of saliva samples and in the use of the smart handheld device. Students who declare themselves to be under psychological or pharmacological treatment will also be included as an important resource to manage their mental health, as these individuals are part of the student reality and can perform adequately from an academic point of view [89–91]. Excluding these individuals could bias the results with respect to the university population as a whole [33]. Furthermore, their inclusion allows for greater representativeness and relevance of the findings, given the current context of mental health in the student population, providing a more complete picture of the impact of academic stress on diverse subpopulations.

#### Exclusion criteria

First-year students and those in their professional practice or internship period will be excluded. Students who decline to participate at any point in the study, who do not comply with the various processes required throughout the study, or who are over 25 years of age will also be excluded.

### Instruments for data collection (Table 2)

**Table 2:** Variables associated with academic performance, their characteristics, and the tools used for their evaluation.

Variable	Description	Measurement Tool	Reliability (Cronbach’s Alpha)
**Academic Stress**	Systemic adaptive process in response to stressors within the academic context.	SISCO-II Academic Stress Inventory	Stressors: 0.78, Physical/Psychological Reactions: 0.89; Social Behavior Reactions: 0.85; Overall: 0.90
**Academic Self-Efficacy**	An individual’s expectations about their ability to organize and perform actions to achieve academic goals.	Academic Self-Efficacy Scale	Attention factor: 0.84, Participation factor: 0.80
**Learning Self-Regulation (LSR)**	Active process through which individuals regulate, control, and monitor cognitions, motivations, and behaviors to achieve academic goals.	LSR Scales	Above 0.70
**Academic Satisfaction**	Perceptions of well-being experienced while performing academic-related activities.	Academic Satisfaction Scale	0.92
**Procrastination**	General tendency to delay the start and/or completion of planned tasks, often accompanied by subjective distress.	Academic Procrastination Scale	0.82 (total scale), 0.75 (activity postponement), 0.82 (academic self-regulation)
**Rumination**	Repetitive thoughts, especially related to depressive rumination, predisposing individuals to maintain and exacerbate depressive symptoms.	Ruminative Response Scale	0.81 (reflective rumination), 0.84 (negative rumination)
**Engagement**	Relationship with intrinsic motivation, positive self-efficacy expectations, optimism, and high self-esteem, which promote academic well-being.	Utrecht Work Engagement Scale-Student (UWES-S)	0.73 (vigor), 0.76 (dedication), 0.70 (absorption)

#### SISCO-II academic stress inventory (SISCO-II)

It is composed of 33 items distributed in three dimensions: Stressors (8 items), Total Reaction (17 items), and Coping Strategies (6 items). The Total Reaction dimension is subdivided into Physical and Psychological Reactions, and Social Behavioural Reactions. Items are scored on a Likert scale from 1 (never) to 5 (always). This instrument has strong psychometric properties with a Cronbach’s alpha of 0.90 and Omega of 0.90 for the full instrument. The Stressors dimension has an alpha of 0.78 and Omega of 0.78, while the Total Reaction dimension shows alpha values of 0.89 for Physical and Psychological Reactions, 0.85 for Social Behavioural Reactions, and Omega values of 0.85 and 0.84, respectively [23,33]. Additionally, norms have been established for university students in Chile, which facilitates the interpretation of the results. For the Stressors dimension, scores below the 40th percentile are classified as low stress, between the 40th and 60th percentiles as moderate stress, and above the 60th percentile as high stress. In the Total Reactivity dimension, scores below the 35th percentile indicate low reactivity, between the 35th and 65th percentiles are considered moderate, and above the 65th percentile are interpreted as high [33]. These norms allow the SISCO-II to provide an accurate and contextualized assessment of academic stress in Chilean university students.

#### Perceived stress scale (PSS14)

Created in 1983 by Sheldon Cohen [92], it measures the degree to which life situations are perceived as stressful and has been adapted for use in the Chilean population, showing internal consistency with a Cronbach’s alpha of 0.80 [34].

#### Academic self-efficacy scale (EACA)

This is a validated instrument in Chile that assesses academic self-efficacy through students’ perceptions of their ability to perform academic tasks [69,93]. The EACA measures the student’s present, ideal, and potential self-efficacy through an assessment of how often the student would perform an action in three time dimensions: present, ideal, and after a conscious effort to improve. The scale is divided into two factors: (i) Attention factor, which assesses the student’s confidence to play a passive role, such as attending to teachers and peers; and (ii) Participation factor, which measures the student’s confidence to play an active role, such as participating in class, completing tasks, and engaging in dialogue with the teacher [69,93]. The scale has demonstrated high reliability, with Cronbach’s alpha values of 0.84 for the Attention Factor and 0.80 for the Participation Factor [94].

#### Self-regulated learning (SRL)

To measure self-regulated learning (SRL) in the academic context, several validated scales will be used to assess the different phases of the self-regulation process. All these scales have demonstrated adequate internal consistency, with Cronbach’s alpha values above 0.70 [36]. (i) Self-Efficacy Scale for Willingness to Study (unidimensional, 7 items): Measures the frequency with which students use strategies to analyse tasks, set goals, and plan their studies. (ii) Study Readiness Scale (unidimensional, 7 items): Assesses beliefs about the ability to use preparatory strategies. (iii) Execution Scale (unidimensional, 16 items): Assesses the frequency of use of monitoring, cognitive, and help-seeking strategies during the performance of academic tasks [36]. (iv) Causal Attributions Scale: Consisting of two subscales, one measuring attributions of failure related to effort and ability (3 items), and the other assesses attributions related to external factors (4 items). Self-Assessment of Study and Learning Planning Scale (unidimensional, 11 items): Measures the frequency with which students evaluate and reflect on the results obtained in academic tasks.

#### Academic satisfaction scale

A widely validated instrument that measures the well-being and enjoyment perceived by students in relation to their learning experiences [73]. The original scale has a single-factor structure, and its validity has been confirmed in Chilean university students through confirmatory factor analysis, showing an adequate fit of the data to the proposed theoretical model. The reliability of the instrument has been assessed using Cronbach’s alpha coefficient, obtaining a value of 0.92, indicating high internal consistency [73,95].

#### Academic procrastination scale (EPA)

An instrument designed to measure both the reasons for and outcomes of perfectionism and procrastination. The EPA assesses two key dimensions: (i) procrastination, measuring the frequency with which students postpone starting or completing academic tasks, and (ii) academic self-regulation, assessing students’ ability to manage and complete academic responsibilities effectively [96,97]. The reliability of the instrument has been validated with a Cronbach’s alpha of 0.82 for the total scale, 0.75 for the procrastination dimension, and 0.82 for the academic self-regulation dimension, which guarantees its accuracy and internal consistency in the assessment of this behaviour [96].

#### Ruminative response scale (RRS)

Developed by Treynor et al. (2003), is a validated instrument that distinguishes between reflective rumination and negative rumination. This scale has been adapted and validated in Chile demonstrating its construct validity to differentiate both forms of rumination in a student population. The scale consists of 14 items, distributed in two subscales: reflective rumination, which has a reliability based on a Cronbach’s alpha of 0.80, and negative rumination, with a reliability of 0.82 [85,98]. This tool allows for an accurate assessment of the tendency to experience repetitive thoughts and their impact on students’ emotional well-being.

#### Well-being in the academic context scale (UWES-S)

It assessing academic engagement and is an instrument consisting of 17 items distributed in three subscales: (i) Vigour, which measures the energy levels and mental disposition of the student to face academic activities; (ii) Dedication, which assesses the degree of involvement and enthusiasm of the student in their studies; and (iii) Absorption, which measures total concentration and sense of immersion in academic tasks [87,99,100]. This scale has demonstrated high internal consistency, with Cronbach’s alpha values of 0.73 for Vigour, 0.76 for Dedication, and 0.70 for Absorption, guaranteeing its reliability for the assessment of academic engagement [87].

### Data collection mechanism

For the study, REDCap will be used as the main platform for data collection and management. REDCap (Research Electronic Data Capture) is a web-based application developed by Vanderbilt University in 2004. Designed to capture clinical research data, REDCap allows the creation and management of surveys and databases in a secure and flexible manner. It is widely used in research institutions around the world due to its Health Insurance Portability and Accountability Act (HIPAA) compliance and its ability to handle sensitive data [101].

The platform allows for the creation of customised forms that are tailored to the specific needs of the study, ensuring the integrity and confidentiality of the data collected. Data capture will be done through electronic surveys distributed to participants via text messages sent to their mobile phones. This will allow participants to access the surveys directly from their smartphone browser, without the need to download additional applications. This approach not only improves accessibility by allowing individuals to complete surveys anywhere, anytime with internet access, but also increases response rates by allowing automatic reminders to be scheduled for those who do not complete surveys. In addition, REDCap offers capabilities for real-time monitoring of participants’ responses, which facilitates accurate tracking and implementation of quality control strategies. This ensures that the data collected is valid and reliable, contributing significantly to the success of the study.

### Use of smart devices

A commercially available wristband-type smart device will be used at a cost of approximately USD 30. This option ensures accessibility and projects the applicability of the project for future initiatives. Although these devices do not offer clinical accuracy in their measurements, their use is suitable for continuously capturing relevant data on biological variables (BADA) from the study, such as heart rate, oxygen saturation and sleep patterns [102,103]. These data will be used to analyse their relationship with the psycho-behavioral variables (PADA) and academic stress, accepting the inherent methodological limitations. Rigorous biosafety protocols will be established to ensure the correct use of the smart devices. These protocols will include specific instructions on proper fitting, maintenance and charging, as well as hygiene recommendations to ensure proper operation during the study.

### Data collection using REDCap and smart devices

To meet the stated objectives, the REDCap platform will be used to manage the collection of data on psycho-behavioral variables (PADA) and academic stress. Students will respond electronically to the instruments designed to measure the variables previously described. The surveys will be conducted three times a week during three evaluation cycles, each of which will last three weeks within an academic semester. The strategic scheduling of the surveys will allow each student to answer only one scale or subscale per day, reducing overload and facilitating continuous participation without interfering with regular academic activities.

Simultaneously, wearable smart devices will be used to collect biological data (BADA) continuously and in real time. These devices will measure heart rate, oxygen saturation, and sleep patterns throughout each academic cycle. The data collected by these devices will be integrated into REDCap, enabling accurate and secure data capture, facilitating further analysis of biological variables associated with academic stress.

### Collection of saliva samples

Participating students will self-collect saliva samples three times per week during each academic year to assess salivary cortisol and amylase levels. Each participant will be trained in the process of self-collection of saliva at home using Salivettes® devices provided by the research team. Students will be instructed in detail on how to collect samples at three specific times: upon awakening (time zero), and then at 30, and 45 minutes. Clear instructions will be provided to ensure proper collection, preservation, and transport of samples, including educational material explaining the procedure. Samples should be kept refrigerated until transport.

For delivery to the laboratory, security and confidentiality protocols will be applied. Samples will be coded to maintain the anonymity of the participants. Once in the laboratory, samples should be processed immediately. They will be centrifuged at 10,000 g for 10 minutes and stored at -20°C until final analysis. This protocol ensures that samples are properly processed and preserved for cortisol and alpha-amylase enzyme activity determinations, applying the relevant biosafety measures.

### Determination of salivary cortisol

Saliva samples will be thawed and quantification will be carried out by ELISA using the commercial Cortisol Saliva ELISA Assay Kit (Eagle Bioscience, CRT32-K01) [104]. Readings will be performed in a microplate reader at a wavelength of 405 nm, with a correction between 570 and 590 nm. For the analysis of the results, the Cortisol Awakening Response (CAR) will be considered, as well as the parameters of area under the increment curve (AUCi) and area under the total curve (AUCg) [44,47,49].

### Determination of salivary alpha-amylase

The detection of salivary alpha-amylase (aAS) enzyme activity shall be performed in saliva samples, using a spectrophotometric method. The procedure is based on the use of 2-chloro-p-nitrophenyl-maltotrioside (CNPG3) as substrate. Alpha-amylase catalyzes the hydrolysis of this compound, releasing 2-chloro-4-nitrophenol, the concentration of which is quantified as a function of the rate of formation. The absorbance of the product is measured at a wavelength of 405 nm, and the catalytic concentration is directly proportional to the alpha-amylase activity in the sample analyzed [105].

### Statistical analysis

First, a descriptive analysis of the variables will be carried out, using measures of central tendency and dispersion for continuous variables, and frequencies and percentages for categorical variables. Next, a bivariate and multivariate analysis will be carried out, comparing the groups according to each variable separately. Subsequently, to analyze the relationship between variables, structural models (SEM) will be used, which are particularly useful for studying the dynamic relationships between different variables over time. In the context of this longitudinal study, cross-lagged random-intercept models will be particularly suitable, as they not only allow assessment of the correlations between variables at different points in time, but also help to understand the directionality of these relationships and the reciprocal effects between key variables, such as academic stress and student performance [106]. This methodological approach is ideal for addressing the hypotheses posed, as it captures how biological and psychobehavioral variables mediate the relationship between academic stress and performance throughout the semester. To assess the mediating effect, confidence intervals will be used for the product of the regression coefficients between the predictor variables and the mediating variable, as well as between the mediating and dependent variables, all calculated using the bootstrap method. The analysis will be performed using the lavaan package in R statistical software, version 4 [107].

### General data plan

At the end of the project, data will be made available to the scientific community through an international open access repository, ensuring transparency and reproducibility of results. A complete general data plan is provided as supplementary material, detailing procedures for data management, storage and protection.

### Ethical considerations

The authors guarantee that all procedures in this research will comply with the ethical standards established by the relevant national and institutional committees on human experimentation, as well as with the principles of the 1975 Declaration of Helsinki, as revised in 2008. The participation of subjects in this study will be voluntary, with the possibility of withdrawing at any time without having to justify their decision. All procedures involving human subjects have been approved by the Scientific Ethical Committee of the Faculty of Medicine of the University of Concepción (N° CEC 4/2024), as well as by the Ethics, Bioethics and Biosafety Committee of the Vice-Rectory of Research and Development of the same university (CEBB 1677–2024). Each participant will be asked to sign an informed consent form, guaranteeing their anonymity and ensuring that they cannot be identified at any stage of the study.

### Publication and dissemination of protocol results

The data obtained in this study will undergo a rigorous process of anonymization prior to publication and/or dissemination, ensuring that all personal or identifiable information is removed. This procedure ensures that the privacy of participants is protected. The anonymised data will subsequently be entered into the University of Concepción’s Dataverse (https://datav.udec.cl/dataverse/udec) in accordance with established protocols for open access to research data. The choice of this Dataverse is due to its robust security infrastructure and its commitment to preserving data integrity.

## Discussion

University life presents a stressful, demanding environment that can affect academic performance and physical and psychological well-being. In addition, students often face financial concerns due to the need to work, dependence on scholarships, family support or indebtedness. There is a lack of longitudinal studies in academia that comprehensively analyze how academic stress affects the well-being and performance of university students. Most research focuses on isolated effects of stressors, without addressing the combined impact holistically. This research seeks to analyze the mediating role of biological and psychobehavioral variables in the relationship between stress and academic performance among undergraduates during a semester. It seeks to answer the question: What is the mediating role of biological and psychobehavioral variables in the relationship between stress and academic performance in university students during a semester? Furthermore, it seeks to understand how these variables influence the relationship and whether they can partially or fully explain the connection between them. In summary, this project examines how biological and psychobehavioral variables mediate the relationship between stress and academic performance in university students during an academic semester.

**Fig 2:**
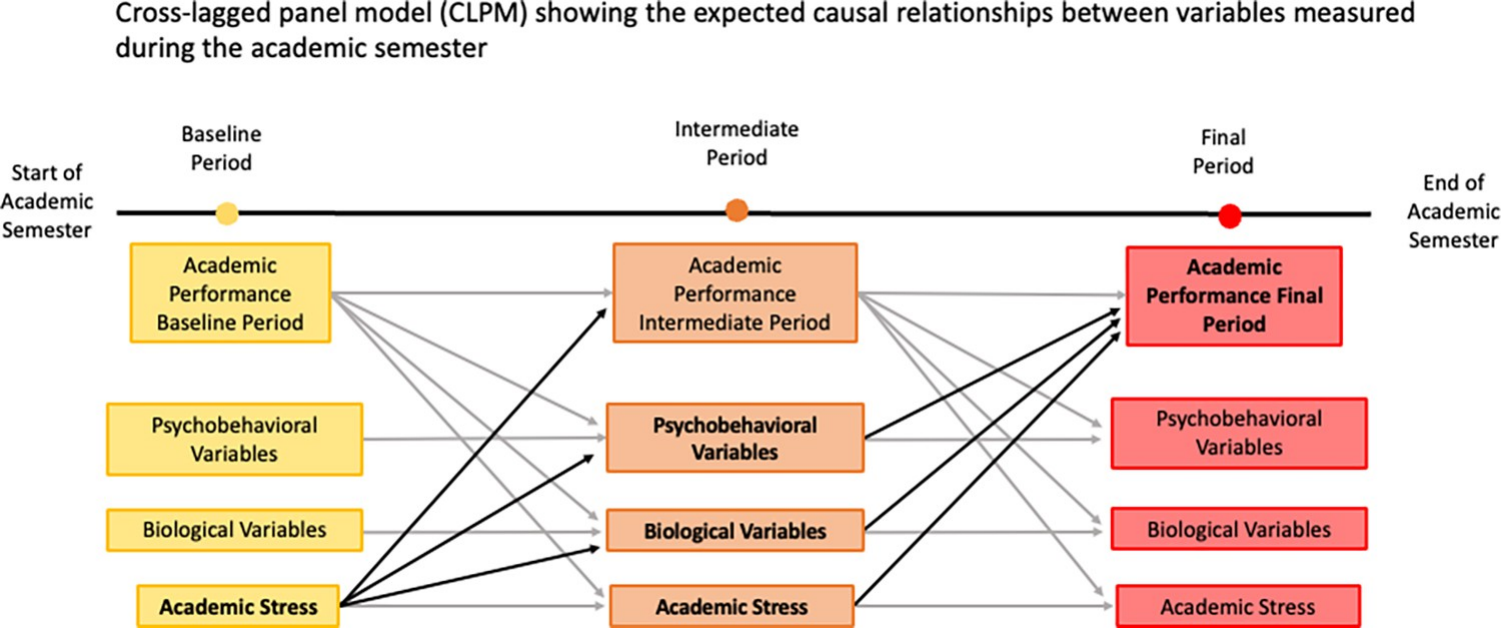
Cross-lagged panel model (CLPM) expected at the end of the semester. The causal relationships expected to be observed at the end of the semester between variables measured during the academic semester are presented.

It is hoped that this research will provide a deeper understanding of the relationship between stress and academic performance in university students, as well as the mechanisms underlying this interrelationship, which could be useful for improving students’ quality of life and academic performance. In this regard, Fig 2 shows a cross-lagged panel model with the expected interrelationship at the end of the semester.

As already discussed, in university students, academic stress and perceived stress can have numerous consequences on students’ physical and mental health, and on their academic performance. It is important to note that these types of stress do not affect all students in the same way, as their impact may depend on various factors, such as personality, social support, financial resources, and level of adaptation to university. However, in physiological terms, this chronic stress can have a negative effect on the immune system, increasing the risk of infectious diseases and other health problems. In addition, it can affect both the sympathetic and parasympathetic nervous systems resulting in increased blood pressure and heart rate [108,109]. In turn, academic stress and perceived stress can decrease motivation, interest, and concentration, which negatively affect academic performance and grades. Moreover, it can contribute to school dropout and disengagement with academic tasks, also affecting short-term memory and hindering decision-making and problem-solving [109,110].

It is essential to bear in mind that, during an academic semester, university students face an increased number of challenges, which affects several aspects of their academic performance, including both physiological factors and those related to the academic environment. Among the physiological aspects affected, those linked to the HPA axis and the ANS stand out. It is therefore crucial to be able to objectively evaluate these changes in order to understand how they influence academic performance.

If we consider that sleep deprivation or lack of adequate rest can cause various disturbances, such as problems with attention, perception, and memory, as well as irritability and stress, it is clear that this affects higher cognitive functions and decreases the ability to perform daily activities effectively [53,54]. In the case of university students, many are forced to sacrifice hours of sleep to fulfill their academic responsibilities, which further increases their stress level. Moreover, even when they do have the opportunity to sleep, they often fail to do so due to their high stress state, creating a vicious cycle between stress and lack of rest. This phenomenon is of particular concern, as stress experienced during the day can significantly alter the quantity and quality of sleep [55,56,111].

On the other hand, the choice of salivary alpha-amylase (sAS) as a biomarker in this study is justified by its role as a sensitive and specific indicator of autonomic nervous system (ANS) activity, particularly sympathetic adrenal-medullary system (SAM) activation [61]. The sAS allows measurement of the ANS response to acute stress, anxiety or chronic stress, as its secretion is significantly increased in response to overactivation of the autonomic system, involving both α-adrenergic and β-adrenergic mechanisms [42]. This aspect is crucial in the context of the research, as it allows for an objective assessment of the impact of stress on university students. Furthermore, the fact that sAS can be measured non-invasively via saliva is a significant advantage, especially when working with sensitive populations, such as students, where obtaining physiological data can be complex [42]. Furthermore, its diurnal profile, with lower levels upon awakening and a steady increase throughout the day, provides dynamic insight into ANS activity in situations of academic stress [42,61]. In chronic situations of ANS dysregulation, the sAS response upon awakening has been found to be attenuated and accompanied by hypersecretion throughout the day, allowing differentiation between typical physiological responses and those associated with a chronic state of stress [61]. The ability to measure sAS with routine clinical methods, such as spectrophotometric or dry chemistry techniques, reinforces its usefulness as a marker in this study, allowing it to be applied in an accessible and repeatable manner to observe changes over time [10,59,62]. For these reasons, the use of the sAS in this project aligns with the need for objective and reliable tools to assess the impact of academic stress on students’ physiological functioning.

In the context of this study, the use of (sAS) as a biomarker not only allows the assessment of autonomic nervous system (ANS) activity, but also provides a window into understanding its relationship with the hypothalamic-pituitary-adrenal (HPA) axis during episodes of academic stress. Studies have shown that sAS responds more rapidly and sensitively to stressful stimuli compared to cortisol, which is particularly relevant for measuring stress peaks that may arise from acute academic situations, such as exams or paper submissions [64,65]. Furthermore, sAS has the advantage of reflecting changes in diurnal rhythm in cases of chronic stress, as observed in patients with post-traumatic stress disorder (PTSD) [66,67]. This altered pattern could have direct implications for the study of the impact of prolonged academic stress, allowing differentiation between typical physiological responses and those indicative of chronic autonomic dysregulation.

Regardless of the effects of stress on the individual, determining academic stress levels is not a minor issue, given the specificity of this concept. In this sense, the use of the SISCO-II Academic Stress Inventory in this study provides a robust and contextualized tool for the assessment of academic stress in university students. Unlike other instruments that have been adapted from general stress contexts, the SISCO-II is specifically designed to address the complexities of stress in the academic setting. This ensures that the data obtained accurately reflect the unique demands and challenges faced by university students, particularly in situations of prolonged stress over the course of an academic semester. The strong psychometric properties of the SISCO-II, with a Cronbach’s alpha and Omega of 0.90 for the full instrument, reinforces its reliability in consistently assessing students’ stressors, physical, psychological, and social behavioral reactions, as well as coping strategies [23]. Additionally, the availability of specific norms for the Chilean population allows for a more accurate interpretation of the results, placing students’ responses in an appropriate context of low, moderate, or high stress. This differentiation is essential to identify subgroups of students who may require specific and differentiated interventions based on their level of stress [33]. With a comprehensive approach that includes both the assessment of stressors and the emotional and behavioral responses to stress, the SISCO-II allows not only to measure the direct impact of stress on academic performance but also to explore the underlying coping mechanisms. This has significant implications for the design of intervention strategies to promote resilience and academic self-efficacy. Therefore, this study not only provides a deeper understanding of how stress manifests in university students, but also offers a solid foundation for future research and academic support programmes.

In turn, the assessment of PADA variables is supported by the appropriate determination of these variables. In this sense, the use of the Academic Self-Efficacy Scale (ASES) in this study will provide an accurate assessment of how students perceive their ability to manage stress and perform academically. As self-efficacy has a considerable influence on students’ effort, persistence, and motivation, this instrument makes a crucial contribution to understanding the impact of academic stress on their performance [69]. The ASES’s ability to measure current as well as ideal and potential self-efficacy provides a comprehensive view of students’ beliefs about their abilities, which is key to assessing not only their current status, but also areas where they could improve with intervention or support. This, combined with its high psychometric reliability, reinforces its relevance as a measurement tool in this protocol [93].

The assessment of self-regulated learning (SRL) in this study will provide a detailed insight into how students manage their learning process, which is fundamental for understanding their academic performance in high-demand contexts. The Study Readiness Self-Efficacy Scale, the Performance Scale, and the Causal Attribution Scale will provide a comprehensive perspective of the cognitive, motivational, and behavioral strategies that students employ to cope with academic demands [36]. These assessment tools will not only help to identify areas where students can improve, but will also provide valuable information to develop educational interventions aimed at optimizing academic performance and strengthening self-regulation skills. A detailed analysis of these variables will be crucial to understanding how academic stress impacts students’ ability to self-regulate their learning and ultimately achieve their academic goals.

The use of the Academic Satisfaction Scale in this study provides a comprehensive assessment of students’ well-being in their academic role, which is crucial to understanding how positive or negative perceptions of their educational environment impact their academic performance. Given that academic satisfaction is linked to academic progress, beliefs in one’s own abilities and academic integration, this instrument will allow for identifying key areas where students experience high or low levels of satisfaction [72]. The high psychometric reliability of the scale, with a Cronbach’s alpha of 0.92, ensures that the results obtained are consistent and representative of the student experience, which in turn provides valuable information for possible interventions aimed at improving academic well-being and, consequently, student performance [73,95].

The use of the Academic Procrastination Scale (EPA) in this study will allow for the identification of procrastination patterns among university students, providing a detailed assessment of how this behaviour affects academic performance and self-regulation. Given that procrastination involves not only inefficient time management but also difficulties in emotional and cognitive self-regulation, the results obtained with the EPA will provide valuable information on how to intervene to improve time management and planning skills in students [79]. Furthermore, the demonstrated reliability of the instrument, with a Cronbach’s Alpha of 0.82 for the total scale, ensures that the data collected is accurate and representative of procrastinative behavior in this context [96,97].

The Ruminative Response Scale (RRS) provides a crucial distinction between the two components of rumination, allowing us to understand how each contributes to students’ emotional well-being in contexts of high academic stress. Negative rumination is associated with more detrimental emotional consequences, and its assessment using the RRS is key to identifying patterns of thinking that may predispose students to depressive symptoms and other emotional difficulties [82,85]. The high reliability of the instrument, with Cronbach’s alphas of 0.80 and 0.82 for the reflective and negative rumination subscales, respectively, ensures that the data obtained is robust and representative, which will contribute to a better understanding of how academic stress may influence students’ mental health [98].

The use of the Well-being in the Academic Context Scale (UWES-S) will allow for an accurate assessment of student engagement, providing key data on levels of Vigor, Dedication, and Absorption, which are fundamental to understanding academic engagement and its relationship to student performance and well-being. The high levels of internal consistency of the scale, with Cronbach’s alphas above 0.70, ensure the reliability of the measurements [87,99]. Assessing academic engagement is essential to identify factors that promote higher performance and well-being, and the results obtained with this scale will contribute to designing strategies to improve students’ engagement and intrinsic motivation.

A key aspect of this proposal is the dissemination of the results. In addition to being shared with the scientific community, information materials on academic stress and its prevention will be developed for university students. These materials will be disseminated through digital platforms and social networks, promoting mental health literacy and encouraging self-care. Digital posters will also be developed to raise awareness among the educational community about the risks of academic stress and coping strategies.

### Limitations

This study has several limitations that need to be considered. Firstly, the methodology used is based on self-report instruments, which could introduce subjective response bias. In addition, despite the ambition of this project, the sample size is relatively small, which could limit the generalizability of the results. Nevertheless, this approach is pioneering in its attempt to integrate biological and psycho-behavioral variables over the course of an academic semester, offering a unique and novel picture of academic stress. It is hoped that future studies with larger sample sizes can replicate and extend these findings. In addition, the study is restricted to university students from a single city, which may limit international comparability.

Another possible limitation of this study is the inclusion of students under psychological or pharmacological treatment, who may have a variable response to academic stress due to the interventions they receive. While this variability can be considered a methodological challenge, its inclusion is essential to capture an increasingly frequent university reality: an increasing number of students face mental health problems and are under treatment. Excluding this group would not only limit the generalizability of the results, but also distort the understanding of the real impact of academic stress in the university environment. Therefore, rather than seeing it as a limitation, we consider that its inclusion strengthens the relevance of the findings. Appropriate statistical methods will be used to control for individual differences and to ensure that the results accurately reflect the impact of academic stress in this population [112–114]. Despite these limitations, the research establishes a solid foundation for future multi-centre collaborations and provides a methodological model for similar research in other populations.

### Strengths

A central strength of this study is the inclusion of students under psychological or pharmacological treatment, which allows for a broader and more representative view of mental health in the student population. This enhances the ecological validity of the study by capturing the diversity of experiences and challenges faced by the university community. In addition, the use of commercial wristbands, which are readily available on the market, facilitates the collection of physiological data in a continuous and non-invasive manner. This not only ensures practicality in implementation, but also opens the door for future studies to replicate and continue this line of research with accessible tools. Finally, the collection of saliva samples, also non-invasive, provides an effective method for measuring biomarkers such as cortisol and alpha-amylase, which minimises stress or discomfort for participants during the process, thus favouring adherence to the study.

## Conclusion

This study represents an opportunity to delve deeper into the mechanisms that mediate the relationship between academic stress and performance in university students, integrating biological and psycho-behavioral variables. By adopting a longitudinal approach and using accessible technologies for data collection, the results will not only provide a solid basis for the design of practical interventions, but also pave the way towards a more comprehensive understanding of the complex interrelationship. between academic stress and the academic environment. These findings could guide the creation of educational policies and support programmes for students in high-stress situations, facilitating the implementation of preventive strategies that promote long-term student well-being. Despite the inherent limitations, this project promises to contribute significantly to knowledge about student coping processes, promoting both well-being and academic success, and laying the groundwork for future studies in this crucial area. In the future, replication of this approach in different academic institutions could strengthen the generalizability of results and allow for the evaluation of personalized interventions.

## Supporting information

S1 File (PDF)
