# Supplementary material for: Longitudinal analysis of academic stress and its effects on salivary cortisol, alpha-amylase, and academic outcomes: Study protocol
Source: PLoS One. 2024 Dec 20;19(12):e0315650. doi: 10.1371/journal.pone.0315650 (PMC11661652; doi:10.1371/journal.pone.0315650)
Supplement: S1 File — (PDF) [file pone.0315650.s001.pdf]

Plan overview Data management*

Administrative Data	
Article title	Longitudinal Analysis of Academic Stress and Its Effects on Salivary Cortisol, Alpha-Amylase, and Academic Outcomes: Study Protocol
Project name	Impact of academic stress on the performance and well-being of university students: An observational longitudinal repeated measures study.
ID	11240383
Funding agency and Grant number	This work will be supported by Grant 11240383 of the National Agency for Research and Development (ANID), Chile, as part of a Fondecyt Initiation project.
Language	English and Spanish
Principal Investigator (PI), identifier and contact details	• Castillo-Navarrete, Juan-Luis; ORCID iD: https://orcid.org/0000-0002-7401-2384; Associate Professor, Departamento de Tecnología Médica, Facultad de Medicina, Universidad de Concepción; Phone: 56998463346, Email: jucastillo@udec.cl ; PhD Programme in Mental Health, Facultad de Medicina, Universidad de Concepción; Programa de Neurociencia, Psiquiatría y Salud Mental, NEPSAM (Neuroscience, Psychiatry and Mental Health programme) (http://nepsam.udec.cl), Universidad de Concepción.
Co-investigators (with identifier)	• Bustos N., Claudio; ORCID iD: https://orcid.org/0000-0003-3478-9858. Email: clbustos@udec.cl<br>• Guzmán-Castillo, Alejandra; ORCID iD: https://orcid.org/0000-0002-0819-4650. Email: aleguzman@ucsc.cl
Project duration	36 months
Project description	• This study investigates the mediating role of biological and psycho-behavioral variables in the relationship between academic stress and performance among university students over the course of a semester. University life often presents a demanding environment, characterized by academic pressures, financial constraints, and personal challenges, which can adversely affect students' academic achievements as well as their physical and psychological well-being. The added burden of balancing work, reliance on scholarships, family support, or managing debt can further compound the stress faced by students. Despite the prevalence of these stressors, there is a notable gap in longitudinal research that comprehensively explores the interaction between academic stressors and their impact on students' physiological and psycho-behavioral responses.<br>• This research addresses the question: *What is the mediating role of biological and psycho-behavioral variables in the relationship between stress and academic performance in university students during a semester?* The goal is to elucidate how these variables influence this relationship and whether they partially or fully explain the connection between academic stress and students' outcomes.<br>• The primary objective is to assess the mediating effects of biological and psycho-behavioral variables on the stress-academic performance relationship during a semester. Understanding these mediating mechanisms will provide insights into the underlying processes that shape this connection. Specifically, the study tests two main hypotheses regarding the mediation roles of biological and psycho-behavioral factors.

	• The research objectives are as follows:<br>(i) To longitudinally assess the levels of academic stress, as well as biological and psycho-behavioral variables associated with academic performance in university students throughout a semester.<br>(ii) To examine the relationship between biological variables and stress levels over the semester.<br>(iii) To explore the relationship between psycho-behavioral variables and stress levels throughout the semester.<br>(iv) To determine the extent to which biological variables mediate the relationship between stress and academic performance.<br>(v) To determine the extent to which psycho-behavioral variables mediate the same relationship.<br>• The study follows a longitudinal observational design, involving repeated measures on students across three evaluation periods, each lasting three weeks, within a semester. Data collection will be managed using the REDCap platform, which will facilitate the administration of electronic questionnaires through a mobile app. This app will assess academic stress and psycho-behavioral variables (PADA). Simultaneously, commercially available smart wristbands will capture continuous data on heart rate, oxygen saturation, and sleep patterns (BADA). The integration of data from the wristbands with REDCap ensures secure and accurate data management throughout the study. Additionally, students will self-collect saliva samples three times per week to measure cortisol and alpha-amylase levels, which will be analyzed using ELISA and spectrophotometry.<br>• Statistical analyses will include descriptive, bivariate, and multivariate approaches, followed by Structural Equation Modeling (SEM) to examine the temporal relationships among variables using a random intercept cross-lagged panel model (RI-CLPM). To assess mediation effects, bootstrap-based confidence intervals will be calculated for the regression coefficients between predictors, mediators, and outcome variables.<br>• In summary, this study aims to deepen our understanding of how biological and psycho-behavioral variables mediate the relationship between stress and academic performance in university students over a semester. The insights gained from this research could inform the development of more targeted interventions to mitigate academic stress and promote better performance and well-being among students.
Date of first version	April 15, 2024
Date of latest version	October 25, 2024

Data collection	
What data will you collect or create?	• **Stress Levels and Psycho-behavioral Variables:** The study will gather data on academic stress levels and psycho-behavioral variables (PADA) using a dedicated mobile app. Students will complete electronic questionnaires three times per week during each evaluation cycle, providing detailed insights into their academic stress levels and related psycho-behavioral responses.<br>• **Biological Variables:** Continuous, real-time data on biological variables associated with academic performance (BADA) will be collected using commercially available smart wristbands. These wristbands will monitor heart rate, oxygen saturation, and sleep patterns throughout the study, providing a consistent stream of physiological data.<br>• **Salivary Biomarkers and Other Physiological Measures:** Students will self-collect saliva samples three times per week to measure levels of cortisol and alpha amylase, which serve as indicators of stress response. These samples will be analyzed using ELISA for cortisol and spectrophotometry for alpha-amylase. The data from these biomarkers will be used alongside wristband measurements to study the interactions between the Hypothalamic-Pituitary-Adrenal (HPA) axis, the Autonomic Nervous System (ANS), and academic stress levels.<br>• All data will be collected longitudinally across three evaluation cycles, each spanning three consecutive weeks within a semester. This approach allows for a comprehensive and dynamic analysis of the relationships between academic stress, physiological responses, and student performance, highlighting both immediate and cumulative effects of stress within the academic context.<br>•
How will the data be collected or created?	• **Mobile Application for Surveys through REDCap:** Students will complete surveys via a custom mobile interface connected to the REDCap platform, three times a week during each evaluation cycle. These surveys will cover aspects of academic stress and psychocomportamental variables (PADA), such as academic self-efficacy, learning self-regulation, academic satisfaction, procrastination, rumination, and engagement. The use of REDCap ensures secure data management and real-time monitoring of survey completion.<br>• **Smart Wristbands for Continuous Data Collection:** Participants will use commercially available smart wristbands to continuously monitor physiological parameters, including heart rate, oxygen saturation, and sleep patterns. These data will be synchronized directly with the REDCap platform, allowing for seamless integration with other collected data and enabling real-time analysis throughout the study period.<br>• **Saliva Sample Collection:** Students will be trained to self-collect saliva samples at specific times: upon waking, and at 30- and 45-minutes post-wake. The collections will take place three times a week during each cycle of measurement. These samples will be analyzed for cortisol and alpha-amylase levels, providing biomarkers of physiological stress response.<br>• **Laboratory Analysis:** Saliva samples will be transported to a laboratory for analysis. Cortisol levels will be measured using the Enzyme-Linked Immunosorbent Assay (ELISA) method, while alpha-amylase activity

	will be assessed via spectrophotometry. The analysis will ensure accurate quantification of these stress biomarkers.<br>• **Data Integration through REDCap:** All data—survey responses, physiological data from smart wristbands, and saliva sample analysis—will be integrated into REDCap's centralized database. This integration allows for a comprehensive longitudinal analysis of the relationships between academic stress, physiological responses, and academic performance, emphasizing the mediation effects of biological and psycho-behavioral variables.
Frequency of new data collection	• **Mobile Application Surveys:** Data on academic stress and psychocomportamental variables (PADA) will be collected during each evaluation cycle using a strategically planned survey distribution. Surveys will be sent at intervals throughout the three-week cycle to avoid overwhelming students with too many questions at once. This approach ensures high response rates while maintaining the well-being of the participants by minimizing the burden of frequent assessments.<br>• **Smart Wristbands:** Continuous monitoring of physiological parameters such as heart rate, oxygen saturation, and sleep patterns will be conducted throughout the entire semester. The smart wristbands provide real-time data, offering a dynamic view of changes in students' physiological states.<br>• **Saliva Sample Collection:** Students will self-collect saliva samples three times per week during each cycle of evaluation. Samples will be taken at specific times—upon waking, and at 30, 60, and 90 minutes after waking—to capture diurnal variations in cortisol and alpha-amylase levels.<br>• **Cycles of Measurement:** Data collection is structured into three cycles, each lasting three consecutive weeks, spaced throughout the semester. This design allows for repeated measures that reflect the evolution of academic stress and related variables, providing a robust dataset for longitudinal analysis.
Quantity of data to be generated	The estimated quantity of data to be generated is approximately 800 to 1000 MB. This estimate encompasses three main sources:<br>• **Continuous Monitoring Data from Smart Wristbands**: With continuous data collection on heart rate, oxygen saturation, and sleep patterns, the wristbands will generate a significant volume of data. Given the number of participants and the duration of data collection over three evaluation cycles, this will contribute substantially to the overall data size.<br>• **Survey Responses**: Data from the mobile application surveys, completed by around 160 participants throughout each evaluation cycle, will generate a robust dataset that includes responses related to academic stress and psychocomportamental variables (PADA). This data will include both qualitative and quantitative elements, adding depth to the analysis.<br>• **Saliva Sample Analyses**: The results from cortisol and alpha-amylase measurements through ELISA and spectrophotometry will add another layer to the dataset. Although the volume of data from each sample analysis is relatively small, the cumulative amount, especially when considering the large number of saliva samples collected (approximately 4,320 samples), will be considerable.<br>The estimated data size of 800 to 1000 MB includes a margin to accommodate potential expansions in data collection or unforeseen increases in the amount of data collected per participant. This comprehensive dataset will allow for an in-depth longitudinal analysis of the relationship between academic stress, biological responses, and academic performance.

Data formats	**Survey Responses:**<br>• **Format:** CSV (Comma-Separated Values) or XLSX (Excel Spreadsheet)<br>• **Purpose:** Used to store responses collected from the mobile application surveys that assess academic stress and psychocomportamental variables (PADA). The chosen formats ensure compatibility with common statistical analysis tools, allowing for straightforward data management and analysis.<br>**Smart Wristband Data:**<br>• **Format:** CSV or JSON (JavaScript Object Notation)<br>• **Purpose:** Captures continuous physiological data such as heart rate, oxygen saturation, and sleep patterns (BADA). The JSON format may be applied when detailed, nested data is collected from wristbands, while CSV provides an accessible format for analysis.<br>**Saliva Sample Analysis:**<br>• **Format:** CSV or XLSX<br>• **Purpose:** Stores numerical data from the analysis of cortisol and alpha-amylase levels. These formats facilitate the integration of biomarker data with other physiological and survey data for comprehensive analysis.<br>**Documentation and Metadata:**<br>• **Format:** DOCX or PDF<br>• **Purpose:** Accompanies the datasets with detailed descriptions of data collection procedures, metadata, and study protocols. These documents ensure transparency and reproducibility, making it easier for future researchers to understand the context and methods used.
What documentation and metadata will accompany the data?	1. **Codebook and Instruction File**:<br>• **Format**: DOCX or PDF.<br>• **Content**:<br>o **Variable Definitions**: Clear explanations of each variable, including those related to psycho-behavioral (PADA) and biological (BADA) factors.<br>o **Coding Schema**: Descriptions of any coding systems used for categorical data, such as stress levels and survey responses.<br>o **Units of Measurement**: Specification of units for numerical data, including heart rate (bpm), oxygen saturation (%), cortisol levels (ng/mL), and alpha-amylase activity (U/L).<br>o **Data Collection Methods**: Detailed procedures for using the mobile app for surveys, continuous monitoring with smart wristbands, and protocols for saliva sample collection.<br>o **Time Stamps and Date Formats**: Explanation of the format for recording dates and times, especially for data from continuous monitoring.<br>2. **Metadata File**:<br>• **Format**: CSV, XLSX, or JSON.<br>• **Content**:<br>o **Dataset Title**: Names of datasets, such as “Survey Responses,” “Wristband Data,” or “Cortisol Levels.”<br>o **Creators**: Details of the researchers responsible for data collection, including their affiliations.<br>o **Date of Creation**: Collection dates and dataset creation timelines.<br>o **Description**: A concise summary of the content and purpose of each dataset.

	o **File Structure**: Information on the dataset's structure, including the number of rows and columns, and a description of each variable.<br>o **Access Conditions**: Restrictions or conditions for accessing the data, including ethical approval or data sharing agreements.<br>o **Version Information**: Updates or revisions to datasets to ensure transparency in data changes over time.<br>3. **Analytical Procedures**:<br>• **Format**: DOCX or PDF.<br>• **Content**:<br>o **Software Used**: Details of software (e.g., R) and versions used for analysis.<br>o **Statistical Methods**: Descriptions of the analytical approaches, including SEM models and cross-lagged panel analysis.<br>o **Quality Assurance Procedures**: Steps taken to ensure data accuracy, such as validation checks, calibration methods, and handling of missing data.<br>**Purpose and Importance**: This documentation and metadata are essential for ensuring that others can understand, interpret, and potentially reuse the data. The comprehensive codebook and metadata files provide a roadmap to the study's dataset, enabling accurate replication of the analyses or integration of the data into future research initiatives.

Ethics and Legal Compliance	
How will you manage the ethical aspects of your research?	**Managing the Ethical Aspects of the Research**<br>**1. Ethical Approvals:**<br>• **The project titled *"Impact of Academic Stress on University Performance and Well-being:*** *A Longitudinal Observational Study"* has received approval from:<br>○ The Faculty of Medicine's Scientific Ethical Committee at the University of Concepción (Approval Code: N° CEC 4/2024).<br>○ The Ethics, Bioethics, and Biosafety Committee of the Vice-Rectory of Research and Development at the University of Concepción (Approval Code: 1172–2022).<br>• These approvals confirm compliance with national and international ethical standards, including the Declaration of Helsinki and Chilean laws on privacy and bioethics.<br>**2. Informed Consent:**<br>• **Informed Consent Process:** Participants will receive comprehensive information about the study's goals, procedures, potential risks, and benefits. A detailed informed consent form will outline this information, divided into sections that provide both the study's description and the formal consent.<br>• **Voluntary Participation:** Participation is entirely voluntary, with no negative consequences for those who decide not to participate or wish to withdraw at any stage of the study.<br>• **Confidentiality:** The informed consent form will describe the measures taken to ensure strict confidentiality, including anonymizing data and using secure, encrypted databases. Participants' identities will be safeguarded, with access to raw data restricted to the principal investigator and authorized research personnel.<br>**3. Data Security and Confidentiality:**<br>• **Data Anonymization:** Participants will be assigned unique codes, and any personal identifiers will be removed from the dataset. Data will be stored securely in encrypted databases, ensuring that participant identities remain protected.<br>• **Access Control:** Only the principal investigator, Dr. Juan Luis Castillo Navarrete, and a limited number of authorized team members will have access to the data, which will be used solely for the purposes of this study.<br>**4. Handling of Sensitive Data:**<br>• **Salivary Biomarker Analysis:** The study involves the collection of saliva samples for cortisol and alpha-amylase analysis. Samples will be processed in specialized laboratories, adhering to strict ethical guidelines. No genetic material will be extracted from the samples.<br>**5. Participant Rights and Protection:**<br>• **Withdrawal Rights:** Participants are free to withdraw from the study at any time, without any need to provide an explanation or fear of consequences.<br>• **Support for Participants:** Should any participant experience psychological distress during the study, they will be provided with resources for mental health support and contact information for relevant services.<br>**6. Publication and Data Sharing:**

	○ **Publication of Results:** The study's findings will be shared in scientific journals and presented at conferences, using only aggregated and anonymized data to ensure participant privacy.<br>○ **Data Sharing:** Any data shared with the research community will be fully de-identified, following the consent provided by participants, ensuring that the data remains secure and respects participant confidentiality.
How will you manage intellectual property rights and copyright issues?	**Managing Intellectual Property Rights and Copyright Issues**<br>1. **Ownership of Data and Research Outputs**:<br>○ The data generated and the research findings, including publications, will be primarily owned by the principal investigator, Dr. Juan Luis Castillo Navarrete, and the University of Concepción. This ownership aligns with institutional policies and those of the funding agency, Agencia Nacional de Investigación y Desarrollo (ANID).<br>○ In cases of significant contributions from external researchers or institutions, co-ownership agreements will be established to clearly define the roles, rights, and responsibilities of each party.<br>2. **Licensing and Usage Rights**:<br>○ The research outputs, such as datasets, will be licensed under open-access licenses like Creative Commons (CC BY-NC). This permits others to share and adapt the work for non-commercial purposes, provided they credit the original authors.<br>○ If third-party data or software is used, all necessary permissions and licenses will be obtained to comply with copyright regulations. Usage conditions will be transparently documented.<br>3. **Publication Rights**:<br>○ When submitting manuscripts for publication, the research team will ensure that agreements align with open-access principles, retaining the right to share preprints or deposit the manuscript in institutional repositories if needed.<br>○ The researchers will retain the right to use their published work for teaching and future research, as long as it aligns with the terms agreed upon with publishers.<br>4. **Data Sharing and Reuse**:<br>○ Anonymized data will be deposited in secure repositories, such as the Dataverse at the University of Concepción (https://datav.udec.cl/dataverse/udec), allowing for controlled access and long-term preservation. Each dataset will be assigned a DOI to ensure proper citation.<br>○ Data shared externally will be subject to agreements specifying proper citation, adherence to ethical standards, and limitations on commercial use, if applicable.<br>5. **Recognition of Contributions**:<br>○ Clear criteria for authorship and contributions will be followed, ensuring that all researchers, including students and collaborators, are properly credited according to ICMJE guidelines. Acknowledgments will reflect the roles of all contributors fairly.

<table>
<tr><th colspan="2">Storage and Backup</th></tr>
<tr><td>How will the data be stored and backed up during the research?</td><td>To ensure the security, integrity, and availability of the data collected during the research, the following measures will be implemented:

Primary Data Storage:

University Servers: All data will be securely stored on password-protected servers managed by the University of Concepción. These servers adhere to institutional security protocols, ensuring compliance with privacy standards.
Data Encryption: Sensitive information, including personally identifiable data, will be encrypted both in transit and at rest using AES-256 encryption protocols, ensuring data confidentiality.

Backup Strategy:

Automated Backups: Data will be backed up daily to minimize data loss risk. Backups will be stored in secure, off-site locations to provide an extra layer of protection against hardware failures or other incidents.
Redundant Storage: Multiple backup copies will be maintained, ensuring data availability in case of corruption or loss of any single backup.
Version Control: Changes to datasets will be tracked through a version control system, allowing restoration of previous data versions if needed, which is essential for longitudinal analysis.

Data Integrity and Disaster Recovery:

Integrity Checks: Regular data integrity checks will be performed to identify and correct any corruption issues. This involves verifying checksums and maintaining consistency across files.
Disaster Recovery: A disaster recovery plan will outline steps for data restoration in case of major incidents, including procedures for restoring from backups and communication protocols for informing stakeholders.

Data Retention:

Long-Term Archiving: After the study's completion, data will be archived in an institutional repository to preserve it for future research and reference.
Retention Policy: Data will be retained according to institutional guidelines, generally for several years. After this period, data will be either anonymized for future use or securely deleted, following ethical requirements.

</td></tr>
<tr><td>How will you manage access and security?</td><td>Managing Access and Security:
To ensure the research data remains secure and access is controlled throughout the study, the following measures will be implemented:

Access Management:

Role-Based Access Control (RBAC): Access will be managed using RBAC, assigning permissions based on the roles of each team member. This ensures that individuals access only the data and tools relevant to their tasks.

Principal Investigator (PI): Full access to all data and administrative functions.

</td></tr>
</table>

	▪ **Research Staff:** Access to specific datasets necessary for analysis, with tailored permissions for editing or viewing as required.<br>▪ **Support Staff:** Limited access, focused on technical tasks like data entry and backup.<br>o **Multi-Factor Authentication (MFA):** MFA will be mandatory for all users, especially for remote access, adding an extra security layer through a code verification system alongside passwords.<br>2. **Data Security Protocols:**<br>o **Data Encryption:**<br>▪ **In Transit:** All data transmitted over networks will be encrypted using SSL/TLS protocols to prevent unauthorized interception.<br>▪ **At Rest:** Data stored on servers or backup devices will be encrypted with standards like AES-256, ensuring it remains secure even in case of unauthorized access.<br>o **Secure Storage:** Data will reside on university-managed servers that meet institutional and industry security standards, with tightly controlled access and monitoring.<br>o **Audit Logs:** Comprehensive logs will track who accesses the data, when, and what actions they perform, aiding in identifying any unauthorized access or activities.<br>3. **Physical Security:**<br>o **Secure Facilities:** Physical servers and backup systems will be housed in secured university data centers with restricted access to authorized personnel.<br>o **Environmental Controls:** These facilities will have temperature regulation and fire suppression systems to protect storage devices physically.<br>4. **Collaboration and Data Sharing:**<br>o **Secure Tools for Collaboration:** Data sharing with external collaborators will use secure tools, like encrypted emails or secure file transfer protocols (SFTP).<br>o **Data Sharing Agreements:** Any external data sharing will be governed by formal agreements that define access terms and confidentiality requirements.<br>5. **Regular Security Reviews and Updates:**<br>o **Security Audits:** Regular audits will assess the effectiveness of security measures, with prompt resolution of any identified vulnerabilities.<br>o **Software Updates:** All software used in data management will be kept updated to protect against known security threats.<br>6. **Incident Response Plan:**<br>o **Reporting and Response:** A protocol for reporting security incidents will be established, ensuring quick notifications to the PI and IT security team in case of breaches.<br>o **Mitigation:** Should a breach occur, affected systems will be isolated, data restored from secure backups, and a thorough investigation conducted to prevent recurrence.<br>7. **Training and Awareness:**<br>o **Team Training:** All research team members will receive training on best practices for data security, including handling sensitive data and recognizing phishing attempts.

	○ **Ongoing Awareness:** Periodic campaigns will keep the team updated on new security threats and reinforce the importance of data protection.

Selection and Preservation	
Which data are of long-term value and should be retained, shared, and/or preserved?	**Which data are of long-term value and should be retained, shared, and/or preserved?**<br>In the context of this research project, the following types of data are considered to have long-term value and will be retained, shared, and preserved:<br>1. **Anonymized Research Data:**<br>o **Type**: Anonymized datasets derived from survey responses (related to academic stress and psycho-behavioral variables), physiological data (e.g., heart rate, blood pressure, sleep patterns), and biological samples (e.g., cortisol and alpha-amylase levels).<br>o **Long-Term Value**: These datasets are valuable for ongoing and future research focused on understanding the relationship between academic stress and student well-being. They can be used for meta-analyses, comparative studies, and as reference datasets for new research projects.<br>o **Retention and Preservation**: These data will be securely stored in a long-term data repository and preserved for a minimum of 5-10 years, or longer as required by institutional and funding agency guidelines.<br>o **Sharing**: Anonymized data will be made available to the scientific community through an open-access data repository, ensuring proper citation and adhering to licensing agreements (e.g., Creative Commons).<br>2. **Processed and Derived Data:**<br>o **Type**: Data that has been processed or aggregated for analysis, including statistical models, correlations, and summarized findings.<br>o **Long-Term Value**: Processed data allows verification of the study's results, supports replication efforts, and serves as a basis for further research. It can also be used to develop educational materials or inform policy decisions.<br>o **Retention and Preservation**: Processed data will be stored alongside the raw data in the repository to ensure accessibility to both forms.<br>o **Sharing**: Processed data will be shared with the raw data, accompanied by documentation that facilitates its understanding and reuse by other researchers.<br>3. **Codebooks and Metadata:**<br>o **Type**: Detailed documentation, including variable definitions, coding schemes, data collection protocols, and assumptions made during analysis.<br>o **Long-Term Value**: Essential for enabling others to understand and interpret the data, making it reusable for future studies.<br>o **Retention and Preservation**: These documents will be stored in PDF or DOCX formats alongside the datasets to ensure long-term accessibility.

	○ **Sharing**: Codebooks and metadata will be openly available with the datasets, ensuring comprehensive understanding of the data structure and context.<br>4. **Research Publications and Reports:**<br>○ **Type**: Final reports, journal articles, conference presentations, and other dissemination outputs.<br>○ **Long-Term Value**: These serve as the primary means of sharing research findings with the academic community and the public, providing a permanent record of the research outcomes.<br>○ **Retention and Preservation**: Publications will be archived in institutional repositories and relevant databases for future reference.<br>○ **Sharing**: Publications will be made available through open-access channels wherever possible, promoting wider dissemination of the research insights.<br>5. **Analysis Scripts and Software:**<br>○ **Type**: Scripts, code, and software used for data analysis, including SEM models and algorithms.<br>○ **Long-Term Value**: Sharing these scripts supports transparency and reproducibility of the research, allowing other researchers to replicate the study or apply the methods to new datasets.<br>○ **Retention and Preservation**: These resources will be preserved in version-controlled repositories like GitHub, ensuring long-term accessibility.<br>○ **Sharing**: Scripts will be made available under open-source licenses, enabling their reuse and adaptation by the scientific community.<br>By retaining, sharing, and preserving these data types, the project ensures its contribution to the broader scientific community, supporting ongoing research, education, and policy-making efforts.
What is the long-term preservation plan for the dataset?	**What is the long-term preservation plan for the dataset?**<br>To ensure that the research data remains accessible, secure, and usable for future studies, the following long-term preservation plan will be implemented:<br>**1. Repository Selection:**<br>○ **Institutional Repository:** The primary dataset will be archived in the University of Concepción's Dataverse repository (https://datav.udec.cl/dataverse/udec), known for supporting secure, long-term data preservation and access.<br>○ **Specialized Repository:** Additionally, the dataset may be deposited in disciplinary repositories like the Open Science Framework (OSF), enhancing visibility within the academic community.<br>**2. Data Preservation Duration:**<br>○ **Retention Period:** Data will be stored for a minimum of 10 years, as required by funding agencies and institutional guidelines, facilitating compliance and future research opportunities.<br>○ **Extended Availability:** Indefinite retention will be considered based on periodic reviews, ensuring continued relevance and accessibility of the dataset.<br>**3. Data Formats and Documentation:**<br>○ **Standard Formats:** Data will be saved in open, non-proprietary formats like CSV for survey and physiological data, and PDF/A or DOCX for documentation.

	- **Comprehensive Metadata:** Detailed metadata, following standards like the Data Documentation Initiative (DDI), will accompany the dataset, providing essential context for future users.<br>**4. Data Integrity and Checks:**<br>- **Regular Checksums:** Routine checksum verifications will be conducted to detect data integrity issues, with protocols for restoring backups if necessary.<br>- **Version Control:** Changes to datasets will be tracked, ensuring that previous versions can be restored if needed.<br>**5. Access and Licensing:**<br>- **Managed Access:** Publicly shareable data will be openly accessible, while any data with ethical restrictions will be managed through controlled access requests.<br>- **Creative Commons Licensing:** Data will be shared under a Creative Commons BY-NC license, allowing reuse while ensuring proper attribution.<br>**6. Redundancy and Backups:**<br>- **Geographical Redundancy:** Multiple copies of the dataset will be stored in different locations to prevent data loss from system failures or natural disasters.<br>- **Cloud Backups:** Cloud storage will provide an additional layer of backup, ensuring data remains accessible even if local systems encounter issues.<br>**7. Periodic Review and Migration:**<br>- **Technology Updates:** The dataset will be reviewed periodically to ensure compatibility with evolving technologies. Data migration to newer formats will be conducted if necessary.<br>- **Relevance Assessment:** The continued relevance of the dataset will be evaluated regularly, and if deemed no longer useful, secure deletion will be carried out in line with institutional policies.<br>**8. Facilitating Data Sharing:**<br>- **DOI Assignment:** A Digital Object Identifier (DOI) will be provided for the dataset, ensuring proper citation and traceability in academic research.<br>- **Promotion of Availability:** Awareness of the dataset's availability will be promoted through academic presentations and publications, encouraging further research and data reuse.

Data Sharing	
How will you share the data?	**How will you share the data?**<br>To ensure that the research data is widely accessible and maximizes its impact, the following data-sharing plan will be implemented:<br>1. **Open Access Availability**:<br>○ **Primary Data Sharing**: Data will be made openly accessible to the public through the institutional repository of the University of Concepción, known as DataV UdeC (https://datav.udec.cl/). Once the data has been collected, processed, and validated, it will be deposited into this repository.<br>○ **Public Access**: The repository provides free access to the datasets, enabling researchers, educators, policymakers, and the general public to benefit from the findings of this study.<br>2. **Repository Details**:<br>○ **DataV UdeC**: This institutional repository offers a secure and reliable platform for data storage, sharing, and long-term preservation. It adheres to international data management standards, ensuring data integrity and accessibility.<br>○ **Metadata and Documentation**: Alongside the datasets, metadata and documentation (e.g., codebooks, methodology descriptions) will be provided to facilitate data understanding, interpretation, and reuse.<br>3. **Licensing and Terms of Use**:<br>○ **Creative Commons License**: Data will be shared under a Creative Commons Attribution-NonCommercial (CC BY-NC) license, allowing others to use, share, and adapt the data for non-commercial purposes, with appropriate attribution to the original authors.<br>○ **Acknowledgment Requirement**: Users must acknowledge the original source in any derivative works, ensuring proper credit to the research team.<br>4. **Data Sharing Timeline**:<br>○ **Immediate Sharing**: Non-sensitive data will be made available as soon as it has been validated.<br>○ **Embargo Period**: Sensitive data or data linked to primary research articles may be subject to an embargo period, after which it will be released for public access.<br>○ **Continuous Updates**: Updates or new data will be uploaded to the repository as they become available, ensuring access to the latest version of the dataset.<br>5. **Access Restrictions (if applicable)**:<br>○ **Sensitive Data Management**: For datasets containing sensitive information, access will be restricted or provided under specific conditions, such as through a request process and adherence to non-disclosure agreements (NDAs).<br>○ **Controlled Access**: The repository will employ controlled access mechanisms to ensure only authorized individuals can access sensitive data.<br>6. **Promotion and Visibility**:

	o **DOI Assignment**: Each dataset will receive a Digital Object Identifier (DOI) to facilitate citation and enhance discoverability in academic literature.<br>o **Dissemination Efforts**: Data availability will be promoted through publications, conference presentations, and outreach to relevant research communities.<br>7. **Feedback and Collaboration**:<br>o **User Feedback**: Users will be invited to provide feedback on their experience, fostering a continuous improvement of data-sharing practices and enabling potential research collaborations.<br>o **Collaborative Opportunities**: Open data sharing aims to foster new research collaborations and joint projects, leveraging the shared datasets for further scientific exploration.
Are any restrictions on data sharing required?	**Restrictions on Data Sharing:**<br>While the aim is to share research data as openly as possible, certain restrictions are necessary to ensure ethical and appropriate use. These restrictions include:<br>1. **Purpose of Use**:<br>o **Scientific and Educational Use Only**: The data will be restricted to use for scientific research and educational purposes. This ensures that the data is applied in ways that align with the original intent of the research, contributing to the advancement of knowledge.<br>o **Prohibition of Commercial Use**: Use of the data for commercial purposes is not allowed. Users must agree not to use the data for profit, consistent with the Creative Commons Attribution-NonCommercial (CC BY-NC) license.<br>2. **Ethical Compliance**:<br>o **Adherence to Ethical Standards**: Users are required to comply with relevant ethical guidelines, including obtaining ethics approval if the data will be used in new studies involving human subjects or sensitive topics.<br>o **Data Anonymization**: The data shared will be anonymized to protect participant privacy. Users must not attempt to re-identify individuals or utilize the data in ways that could compromise confidentiality.<br>3. **Attribution and Acknowledgment**:<br>o **Proper Citation**: Users are required to properly cite the source of the data in publications, presentations, or other derivative works. This ensures that the research team's contributions are acknowledged.<br>o **No Misrepresentation**: The data must not be used in ways that distort the findings or misrepresent the original research.<br>4. **Data Access Agreements**:<br>o **User Agreements**: Access to certain sensitive datasets may be provided only under specific agreements, such as non-disclosure agreements (NDAs) or confidentiality clauses.<br>o **Review of Access Requests**: Requests for access to restricted data will be reviewed by the research team or an ethics committee to ensure that the intended use aligns with the project's ethical and legal standards.<br>5. **Embargo Periods** (if applicable):<br>o **Temporary Restrictions**: Certain datasets may be under an embargo period, allowing time for the publication of primary

	research findings. After this period, data will be made available with the outlined restrictions.<br>6. **Monitoring and Enforcement**:<br>o **Monitoring of Use**: The research team may monitor the use of the data to ensure compliance with the terms and conditions. This may include periodic checks of publications or projects citing the data.<br>o **Enforcement of Restrictions**: In cases of misuse or violations, the research team reserves the right to revoke data access and take appropriate actions, such as notifying authorities or retracting access rights.

Responsibilities and resources	
Who will be responsible for data management?	**Roles and Responsibilities for Data Management**<br>To ensure effective and secure data management throughout the research project, the following roles and responsibilities have been defined:<br>**1. Principal Investigator (PI):**<br>○ Name: Dr. Juan Luis Castillo Navarrete; ORCID iD: https://orcid.org/0000-0003-3478-9858; Email: jucastillo@udec.cl<br>○ **Role:** The Principal Investigator (PI) will have overall responsibility for data management in the project. Dr. Castillo Navarrete will oversee all aspects of data collection, storage, analysis, sharing, and preservation.<br>○ **Responsibilities:**<br>▪ Ensuring compliance with institutional, ethical, and legal standards for data management.<br>▪ Overseeing the secure storage and regular backup of all research data.<br>▪ Coordinating the submission of data to the institutional repository (DataV UdeC) for long-term preservation and public access.<br>▪ Managing access control to ensure that only authorized personnel can access sensitive data.<br>▪ Reviewing and approving data sharing agreements, ensuring that conditions for access to restricted data are respected.<br>▪ Facilitating training sessions for the research team on best practices for data handling, security, and confidentiality.<br>**2. Co-Investigators:**<br>○ Name: Claudio Bustos N.; ORCID iD: https://orcid.org/0000-0003-3478-9858; Email: clbustos@udec.cl<br>○ **Name:** Alejandra Guzmán-Castillo; ORCID iD: https://orcid.org/0000-0002-0819-4650; Email: aleguzman@ucsc.cl<br>○ **Role:** Both co-investigators will support the PI in ensuring effective data management practices and will collaborate in overseeing data collection, analysis, and storage.<br>○ **Responsibilities:**<br>▪ Assisting in the development and implementation of data collection protocols.<br>▪ Monitoring data integrity and quality during the research process.<br>▪ Reviewing data anonymization processes and ensuring the correct documentation is in place for data sharing.<br>▪ Supporting the preparation and submission of datasets to the institutional repository, ensuring that metadata is complete and accurate.<br>▪ Collaborating with the PI in managing access requests for sensitive data, ensuring that all conditions of use are met.<br>**3. Data Manager:**

- **Role:** The Data Manager will support the PI and co-investigators in handling day-to-day data management tasks, ensuring data quality and security.
- **Responsibilities:**
  - Implementing data collection protocols and monitoring data integrity during the research process.
  - Managing the infrastructure for data storage, including encrypted storage solutions and coordinating daily backups.
  - Preparing datasets for submission to the institutional repository, focusing on data anonymization and proper documentation.
  - Conducting regular checks of access logs to identify any unauthorized access attempts.
  - Collaborating with the PI and co-investigators to address any data breaches or anomalies promptly, ensuring data security is maintained.

4. **Research Team Members:**
   - **Role:** All research team members involved in data collection, analysis, and storage will adhere to established data management protocols.
   - **Responsibilities:**
     - Collecting and recording data according to established protocols and ethical guidelines.
     - Ensuring accurate recording and secure storage of data, with proper labeling and organization.
     - Reporting any discrepancies, data anomalies, or security concerns to the Data Manager, PI, or co-investigators without delay.
     - Participating in mandatory training sessions on data security and ethical handling of sensitive information.
5. **Institutional Support:**
   - **Role:** The University of Concepción's IT department and DataV UdeC team will provide technical support for data management.
   - **Responsibilities:**
     - Maintaining secure servers and backup systems that comply with institutional and industry standards.
     - Assisting with encryption of sensitive data and providing technical safeguards for data access.
     - Managing the DataV UdeC repository, including metadata management and the long-term preservation of datasets.
     - Offering guidance on data management practices, ensuring compliance with institutional policies.
6. **Ethics and Compliance Officer:**
   - **Role:** The Ethics and Compliance Officer will ensure alignment of data management practices with ethical standards set by the University of Concepción and relevant regulations.
   - **Responsibilities:**
     - Reviewing the data management plan to ensure adherence to ethical guidelines.

	▪ Monitoring compliance with data protection regulations to maintain participant confidentiality.<br>▪ Assisting with the preparation and review of informed consent documents related to data sharing, ensuring transparency with participants.
What resources will you require to develop your plan?	**Resources Required for Data Management Plan Implementation**<br>To successfully develop and implement the Data Management Plan (DMP) for this research project, the following resources will be necessary:<br>1. **Technical Resources:**<br>o **Secure Data Storage Solutions:**<br>▪ **Servers:** Access to secure, high-capacity servers provided by the University of Concepción for storing research data. These servers should support encryption, regular backups, and secure access controls to maintain data integrity and security.<br>▪ **REDCap Platform:** The REDCap system will facilitate survey data collection and organization, providing a user-friendly interface for storing psychocomportamental data collected from students.<br>▪ **Cloud Storage (Optional):** Cloud services like Dropbox or OneDrive may be used as an additional layer of redundancy, offering remote access and enhancing the project's flexibility in data access.<br>o **Backup Systems:**<br>▪ **Automated Backup Software:** Software for automated and regular data backups to ensure data preservation. This includes the ability to restore data in case of unexpected loss or corruption.<br>o **Data Encryption Tools:**<br>▪ **Encryption Software:** Advanced encryption software like AES-256 will be used to secure sensitive data during storage and transfer, ensuring data protection against unauthorized access.<br>2. **Software and Applications:**<br>o **Data Analysis Software:** Statistical tools such as R, SPSS, or Python will be required for processing and analyzing data, including running Structural Equation Models (SEM) and cross-lagged analysis.<br>o **ELISA and Spectrophotometry Equipment:** For the laboratory analysis of salivary cortisol and alpha-amylase, access to ELISA readers and spectrophotometers is essential.<br>o **Version Control Systems:** Tools like Git for version control will manage updates to data processing scripts, ensuring that previous versions can be restored if needed.<br>o **Secure Collaboration Tools:** Platforms like encrypted cloud services or secure file transfer protocols (SFTP) will support data sharing among team members while maintaining data security.<br>3. **Human Resources:**<br>o **Data Manager:** A dedicated Data Manager will oversee the management, storage, backup, and sharing of data, ensuring data integrity throughout the study.

	- **IT Support Staff:** The University of Concepción's IT team will support the setup and maintenance of servers, encryption protocols, and REDCap.<br>- **Training for Research Team:** Workshops on data management best practices, including data security and ethical handling, will equip team members with the necessary skills.<br>- **Ethics and Compliance Officer:** Assistance from the Ethics and Compliance Officer will ensure that all data management practices adhere to ethical standards and regulations.<br>4. **Financial Resources:**<br>- **Budget for Technical Infrastructure:**<br>- **Server and Storage Costs:** Funding for maintaining secure server access and cloud storage services.<br>- **Software Licenses:** Costs for statistical software and encryption tools.<br>- **Personnel Costs:** Funding for the roles of the Data Manager, IT support, and training sessions for the research team.<br>- **Laboratory Costs:** Budget for reagents and consumables needed for analyzing biological samples.<br>5. **Institutional Support:**<br>- **Institutional Repository Access:** Support from the University of Concepción's DataV UdeC repository for long-term data storage, ensuring compliance with open-access mandates.<br>- **Ethical Review and Guidance:** Continuous guidance from the University's ethics committee will ensure compliance with ethical standards in data handling.<br>6. **Documentation and Guidelines:**<br>- **Data Management Policies:** Access to the University of Concepción's data management guidelines and funding agency requirements (e.g., ANID).<br>- **Best Practice Guides:** Reference materials on data curation from sources like the Digital Curation Centre (DCC) and Data Documentation Initiative (DDI) to ensure high-quality data management.

(*) Adapted from:

- 2.3 Componentes de un PGD - Gestión de datos de investigación - Biblioguias at Biblioteca CEPAL, Naciones Unidas [Internet]. Available from: https://biblioguias.cepal.org/gestion-de-datos-de-investigacion/ComponentesPGD
- UdeC. Plan de Gestión de Datos [Internet]. Plan de Gestión de Datos. Dirección de Bibliotecas. 2022. Available from: https://bibliotecas.udec.cl/wp-content/uploads/2022/08/Plan_Gestion_Datos-UdeC.pdf
- DCC. Checklist for a Data Management Plan, v4.0 [Internet]. 2013. Available from: https://dmponline.dcc.ac.uk/files/DMP_Checklist_2013.pdf
